# Kea (*Nestor notabilis*) show flexibility and individuality in within-session reversal learning tasks

**DOI:** 10.1007/s10071-021-01524-1

**Published:** 2021-06-10

**Authors:** Monika Laschober, Roger Mundry, Ludwig Huber, Raoul Schwing

**Affiliations:** 1 Comparative Cognition, Messerli Research Institute, University of Veterinary Medicine Vienna, Medical University of Vienna, University of Vienna, Vienna, Austria; 2 Platform Bioinformatics and Biostatistics, University of Veterinary Medicine Vienna, Vienna, Austria

**Keywords:** Midsession reversal, Win-stay/lose-shift, Reversal estimation, Parrot cognition, GLMM, Touchscreen

## Abstract

The midsession reversal paradigm confronts an animal with a two-choice discrimination task where the reward contingencies are reversed at the midpoint of the session. Species react to the reversal with either *win-stay/lose-shift*, using local information of reinforcement, or *reversal estimation*, using global information, e.g. time, to estimate the point of reversal. Besides pigeons, only mammalian species were tested in this paradigm so far and analyses were conducted on pooled data, not considering possible individually different responses. We tested twelve kea parrots with a 40-trial midsession reversal test and additional shifted reversal tests with a variable point of reversal. Birds were tested in two groups on a touchscreen, with the discrimination task having either only visual or additional spatial information. We used Generalized Linear Mixed Models to control for individual differences when analysing the data. Our results demonstrate that kea can use win-stay/lose-shift independently of local information. The predictors group, session, and trial number as well as their interactions had a significant influence on the response. Furthermore, we discovered notable individual differences not only between birds but also between sessions of individual birds, including the ability to quite accurately estimate the reversal position in alternation to win-stay/lose-shift. Our findings of the kea’s quick and flexible responses contribute to the knowledge of diversity in avian cognitive abilities and emphasize the need to consider individuality as well as the limitation of pooling the data when analysing midsession reversal data.

**Supplementary Information:**

The online version contains supplementary material available at 10.1007/s10071-021-01524-1.

## Introduction

An animals’ environment is not necessarily stable over time. Living in demanding and constantly changing environments may therefore select for individuals with cognitive abilities that enable them to be successful even in variable situations (Milton 1981; Bond et al. 2007; Shettleworth 2010). Over the last decades researchers had used a variety of reversal learning tasks to assess how flexible animals react when the contingencies of a task reverse to the opposite at some point within the test (Bitterman 1965; Mackintosh 1974). In such tests the animals’ flexibility, the promptness with which they are able to adapt to the new contingency, is seen as a measure for their ‘intelligence’ (Bond et al. 2007). In serial reversal tests, reversals happen repeatedly between each session (e.g. Bond et al. 2007). Typically, animals are tested only once a day, requiring that the animal remembers the last correct stimulus over several hours or even days between two sessions, therefore memory has an influence on the animals’ responses and may interfere with the learning ability or behavioural flexibility of the subject under investigation (Cook and Rosen 2010).

To avoid the memory influence, researchers adopted a midsession reversal paradigm (MSR), which tests the animal’s immediate response to the reversal of reinforcement contingencies at the midpoint of each session (Cook and Rosen 2010; Rayburn-Reeves et al. 2013b). As the reversal is always at the same point within the session, after multiple repetitions, test subjects use one of two response patterns or strategies to deal with the potentially predictable reversal: *win-stay/lose-shift* or *reversal estimation*. The reversal estimation (Cook and Rosen 2010; Rayburn-Reeves and Cook 2016) relies on global information—for example the time or the number of trials into the session—to estimate the point of reversal. When adopting this strategy, test subjects are prone to commit two kinds of ‘estimation errors’ around the reversal called *anticipatory* and *perseverative* errors, representing anticipation of the second correct stimulus when still incorrect or perseverating the choice of the first correct stimulus although already incorrect. The win-stay/lose-shift strategy (Cook and Rosen 2010; Rayburn-Reeves and Cook 2016) avoids these errors as it uses the local information of the previously reinforced stimulus to either continue with the previous response as long as it is being rewarded or to switch to the other if suddenly a reward fails to appear.

Previous research has found that the test setup of the underlying discrimination task, as well as the species tested, influences which response to the reversal is shown. Humans (Cook and Rosen 2010; Rayburn-Reeves et al. 2011; but see McMillan and Spetch 2019) as well as rats (Rayburn-Reeves et al. 2013b, 2018; but see Smith et al. 2016) and macaques (Rayburn-Reeves et al. 2017) tend to adopt win-stay/lose-shift, while pigeons (e.g. Rayburn-Reeves et al. 2011, 2013b; McMillan and Roberts 2012; McMillan et al. 2016) and dogs (Laude et al. 2016) usually exhibit reversal estimation. Pigeons were shown to use the time into the session (McMillan and Roberts 2012, 2015; Daniel et al. 2015) rather than satiety levels (Cook and Rosen 2010) or the number of trials for their estimation (Rayburn-Reeves and Cook 2016). Tasks with a shifted reversal position (Rayburn-Reeves et al. 2011, 2013a, b; McMillan et al. 2014) were used to encourage, however insufficiently, the pigeons to switch to the use of the local information of reinforcement. Pigeons used win-stay/lose-shift only in tests with a short inter-trial-interval of 1.5 s and when spatial information was the discrimination cue in the task (Rayburn-Reeves et al. 2013a).

It could be assumed that behaviour varies within individuals over time as well as between individuals of the same species. Still, previous studies analysed the data pooled over subjects and over the last 10–20 sessions, which does not allow to consider the individual responses to the reversal per session. Additionally, several mammalian species were tested in a midsession reversal paradigm, but pigeons were the only bird species tested so far. Besides pigeons and corvids, parrots are often used in avian cognition studies (Pepperberg 1999; Emery 2006; Cussen 2017; Auersperg and von Bayern 2019). Parrots recommend themselves for this study paradigm due to their life history and the variety of ecological problems they face, as well as due to their neuroanatomy (Emery 2006; Cussen 2017). Here we tested kea (*Nestor notabilis*), New Zealand’s alpine parrots, known for their explorative and neophilic behaviour (O’Hara et al. 2012, 2017). Their responses in the described paradigm are interesting as, although phylogenetically closer related to pigeons, kea are comparable to rats and humans, in other aspects. Kea have a diverse feeding ecology (Jackson 1960; Schwing 2010; Greer et al. 2015), including a variable, omnivorous diet and different feeding styles (Marriner 1906; Breejart 1988; Diamond and Bond 1999; Beggs and Mankelow 2002; Juniper and Parr 2003; Young et al. 2012), an elongated juvenile period and live in fission–fusion social systems (Diamond and Bond 1999). Kea also have proved themselves as extremely flexible in behavioural and cognitive terms by quickly switching between different problem-solving strategies even after one has proved to be successful (Werdenich and Huber 2006) or by spontaneously innovating tool use when other attempts to reach encapsulated food failed (Auersperg et al. 2011a, b). These aspects could possibly influence the responses in the task. A pilot study also suggested individual differences in learning the task and the responses to the reversal in a fully randomized visual task. Here, we therefore aimed to explore the kea’s response strategies in within-session reversal paradigms with regard to an effect of task requirement, with consideration of possible individual differences between birds and between sessions of individual birds. Additionally, we wanted to not only address the disadvantages of pooled data, but also provide a possible solution.

**Fig. 1:**
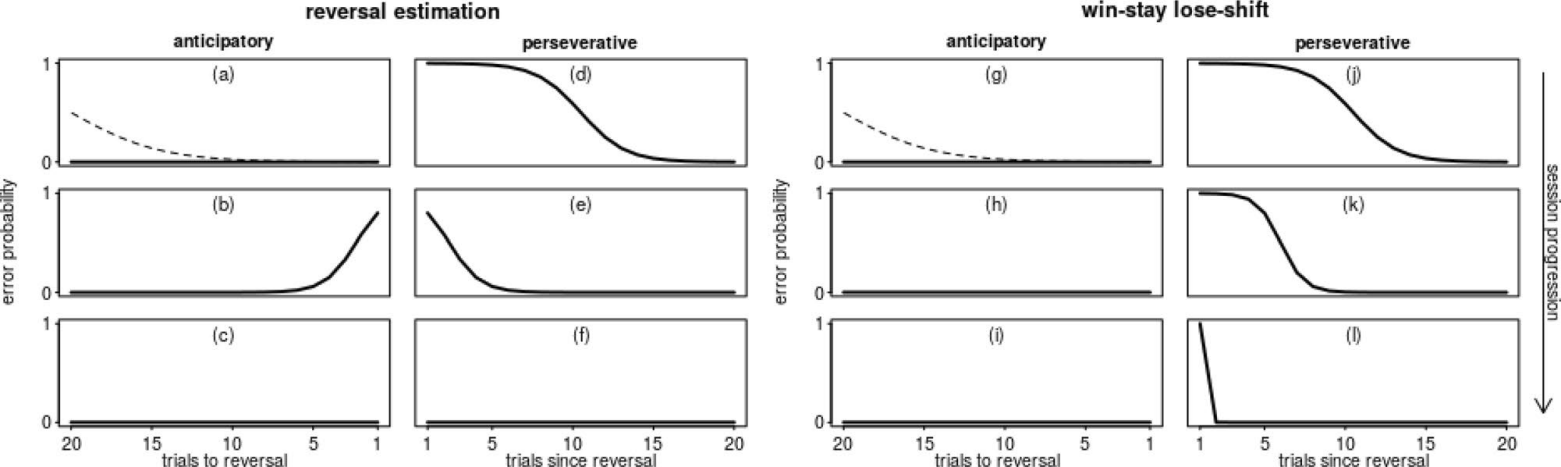
Expected probabilities of anticipatory (**a**–**c** and **g**–**i**) and perseverative (**d**–**f** and **j**–**l**) errors for animals using a perfect reversal estimation strategy (**a**–**f**) and a perfect win-stay/lose-shift (**g**–**l**) strategy and how it evolves over the course of the experiment (top to bottom per column). In the first session, when animals had never experienced a reversal, errors do rarely occur before the reversal (**a** and **g**) and after the reversal their probability slowly decreases as animals learn to adjust their choices to the newly rewarded stimulus (the dashed line shows a hypothetical development for animals which are initially naïve). As the sessions progress (top to bottom), an animal using a reversal estimation strategy will tend to exhibit more anticipatory errors towards the end of the period before the reversal (**b**) but also commit errors regularly after the reversal (**e**). An animal that perfectly manages a reversal estimation strategy will eventually be able to switch their choice at the trial at which the reversal takes place and not commit any errors, neither before (**c**) nor after (**f**) the reversal. Animals using a win-stay/lose-shift strategy are never expected to commit anticipatory errors with an elevated probability (**g**–**i**) but will gradually reduce the probability perseverative errors in all trials except the very first one after the reversal (**j**–**l**)

We analysed the occurrence of anticipatory and perseverative errors in a within-session reversal learning paradigm. When the individuals use reversal estimation, errors should become more likely towards the shift, and when the individuals use a win-stay/lose-shift strategy, the probability of errors should not increase towards the reversal, but right after it and rapidly decrease again (Fig. 1). Moreover, when individuals improved their reversal estimation or changed strategies over the course of sessions, these predicted effects should become less or more pronounced, respectively, over the course of the experiment. Furthermore, estimating these effects in the framework of hierarchical models allows one to estimate the amount of between- and within-individual variation in the strategy used.

## Methods

### Study animals

We tested twelve adult kea (*Nestor notabilis*), six of them females, aged between 3 and 18 years from April to July 2017. The kea were kept in a group of 23 birds in a spacious and environmentally enriched outdoor aviary. The birds were fully fed on a diverse diet with free access to water and remained at the research station after data collection.

### Apparatus

The kea were tested individually in an outdoor testing compartment, on a touchscreen device. The touchscreen apparatus consisted of a 38.7 × 50 × 60.8 cm (w × h × l) metal box with a 15-inch infrared touch frame (‘CarrollTouch’ D87587-001) mounted in front of a 15-in. XGA colour TFT computer screen on its front side (see Steurer et al. 2012 and O’Hara et al. 2015 for more information). The birds’ feeding tray, was located centrally below the touchscreen. The touchscreen and an automatic feeding system were connected via a personal computer. The software CognitionLab (CogLabLight 1.9; © 2008 Michael Morten Steurer) ran the experiment and controlled the feeder. All but one kea had touchscreen experience. The inexperienced bird had six touchscreen training sessions unrelated to the task, before testing. Seven birds had taken part in our pilot study on midsession reversal but had not learned either of their correct stimuli and had shown a side bias throughout the experiment instead. Eight birds had experience in at least one other variant of a reversal paradigm (see Stobbe et al. 2012; O’Hara et al. 2015; Wein et al. 2015).

### Procedure

Birds were tested in the midsession reversal task by giving them a two-choice discrimination task. As stimuli we used simple computer images of a yellow drop and a turquoise star, each fitting into a 12 cm^2^ black square (see Laude et al. 2016) and presented on a black background. The stimuli were located at the bottom half of the screen, 18 cm apart (measured from the centre of the stimulus) and 6 cm from the lower and the side rim. Birds were tested in two groups (visual, visuo-spatial) with visual information only (stimuli changed the location on the screen semi-randomly, with a maximum of three consecutive presentations on the same side) or with additional spatial information (stimuli remained on the same side of the screen throughout the whole experiment) for the discrimination. Each bird participated in 60 midsession reversal sessions, with 40 trials per session and the reversal at trial 21, based on a similar setup in a study with pigeons and dogs (Laude et al. 2016). The choice of the positive stimulus was indicated by a sound (600 Hz, 100 ms long) and reinforced with a piece of peanut, then the stimuli disappeared. The response to the negative stimulus induced a different sound (200 Hz, 200 ms long) but had no consequences for the birds except that it also terminated the stimulus presentation. Intertrial interval was two seconds with an additional second for feeding when positive. For an additional eight sessions, birds were confronted with a shifted reversal either at trial 11 or 31 alternated with a normal reversal session (i.e., at trial 21). In each group, half of the birds had their first session with an early reversal, the other birds with a late reversal. Side and type of the reinforced stimulus were counterbalanced in each group.

### Statistical analysis

To model the error probability we fitted a total of four Generalized Linear Mixed Models (GLMM; Baayen 2008), one for each combination of the type of error (anticipatory and perseverative) and reversal timing (midsession or shifted reversal). For an overview of model parameters see Table 1, for more detailed information on models and methods of analysis please see the supporting information (SI_1). The response was the occurrence of an error at the individual trial, and hence we fitted the model with binomial error structure and logit link function (McCullagh and Nelder 1989). Key fixed effects were *trial number*, *session number* (linear and additionally squared for models 1 and 3), and *group* (visual or visuo-spatial), and all their interactions up to the third order. Additional fixed effects were *age* and *sex*.

**Table 1:** Details on the four GLMM models and their terms

	Model 1	Model 2	Model 3	Model 4
Test	Midsession	Midsession	Shifted	Shifted
Part	Bevor reversal (anticipatory errors)	After reversal (perseverative errors)	Bevor reversal (anticipatory errors)	After reversal (perseverative errors)
Response	Occurrence of errors at individual trials	Occurrence of errors at individual trials	Occurrence of errors at individual trials	Occurrence of errors at individual trials
Error structure	Binomial error structure and logit link function	Binomial error structure and logit link function	Binomial error structure and logit link function	Binomial error structure and logit link function
Key fixed effects^a^	Session number (linear and squared), trial number, group	Session number (linear), trial number, group	Session number (linear and squared), trial number, group	Session number (linear), trial number, group
Further fixed effects	Age and sex	Age and sex	Age and sex	Age and sex
Random intercept effects	Individual, session ID nested within individual	Individual, session ID nested within individual	Individual, session ID nested within individual	Individual, session ID nested within individual
Random slopes	Trial number within session	Trial number within session	Trial number within session	Trial number within session
	Session number (linear and squared), trial number and their interaction within individual	Session number (linear), trial number and their interactions within individual	Session number (linear and squared), trial number and their interaction within individual	Session number (linear), trial number and their interaction within individual
Sample size	14,400 trials 12 individuals 720 sessions	14,400 trials 12 individuals 720 sessions	1,920 trials 12 individuals 96 sessions	1,920 trials 12 individuals 96 sessions

We z-transformed *trial number*, *session number*, and *age*

^a^We also included all their interactions up to order three

We included random intercept effects (see Table 1) to model variation among individuals and from session to session as well as to avoid pseudo-replication. We included random slopes (see Table 1; Schielzeth and Forstmeier 2008; Barr et al. 2013) to keep type I error rate at the nominal level of 0.05 as well as to be able to infer about strategies varying between individuals and/or from session to session within individuals. Correlations among random intercepts and slopes were removed from the model in case they appeared in part unidentifiable (as indicated by many absolute correlation parameters estimated as essentially 1; Matuschek et al. 2017). Please see the supporting information for the initial and final full models fitted. To avoid 'cryptic multiple testing' (Forstmeier and Schielzeth 2011) we compared each full model with a respective null model lacking the key fixed effects in the fixed effects part but being otherwise identical.

For the purpose of exploring the magnitude of variation among and within birds, we first compared the standard deviation estimated for the contribution of the random effects with the value of the respective fixed effect. Such a comparison is possible since both indicate the influence of the respective effect on the response on the same scale (“link space”; McCullagh and Nelder 1989). Second, we extracted *Best Linear Unbiased Predictors* (BLUPs; Baayen 2008) and plotted the estimated individual specific effects together with group level effects and the individual observations to get an impression about the magnitude of variation among and between individuals.

We fitted the models in *R* (version 3.6.3; R Core Team 2020) using the function glmer of the package lme4 (version 1.1–21; Bates et al. 2015) utilizing the optimizer 'bobyqa'. Prior to fitting the models, we z-transformed *trial number*, *session number*, and *age* to ease model convergence and achieve easier interpretable model coefficients (Schielzeth 2010). We conducted full-null model comparisons by means of likelihood ratio tests (Dobson 2002) and tested individual fixed effects by dropping them from models one at a time and utilizing a likelihood ratio test to compare the simpler with the more complex model (Barr et al. 2013). The stability of the full models was estimated to be of moderate to good stability (see results, for more details see the supporting information SI_1). We determined confidence intervals of the model estimates and fitted values by means of a parametric bootstrap (*N* = 1000 bootstraps; function bootMer of the package lme4). In case of the shifted reversal experiments (models 3 and 4) we aligned trial number according to when the reversal occurred; that is, in all sessions the last trial before the reversal got the same trial number, and the first trial after the reversal got the same trial number, too. All Figures were created in R (version 3.6.3; R Core Team 2020).

## Results

### Overview of pooled data

**Fig. 2:**
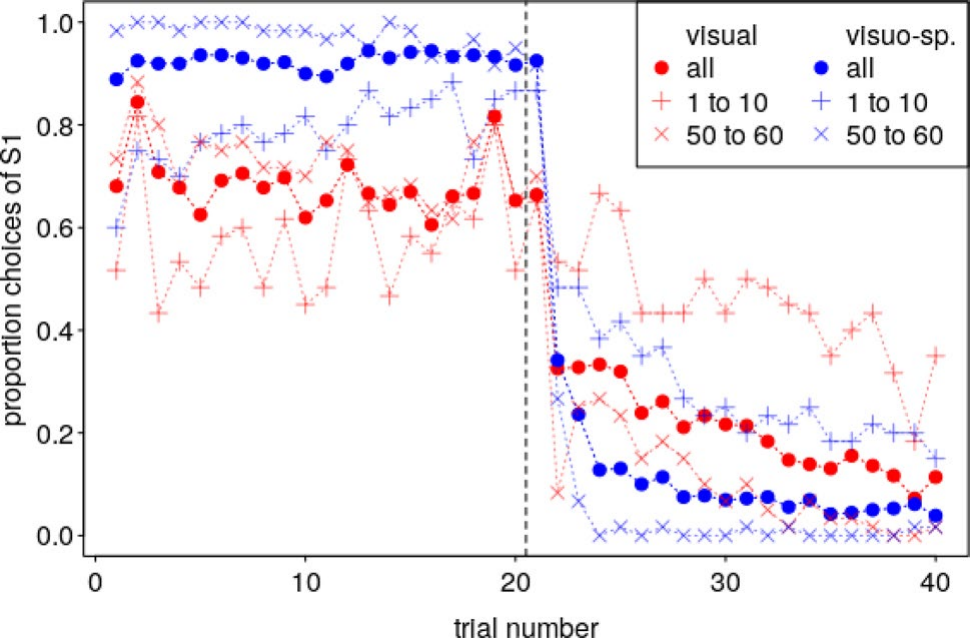
Proportion of trials in which the birds chose the first correct stimulus (S1) in the midsession reversal experiment. Note that this proportion was still high in the first trial after the reversal (vertical dashed line) but then steeply decreased, particularly in visuo-spatial group. Note also that the visuo-spatial group performed better than the visual group

**Fig. 3:**
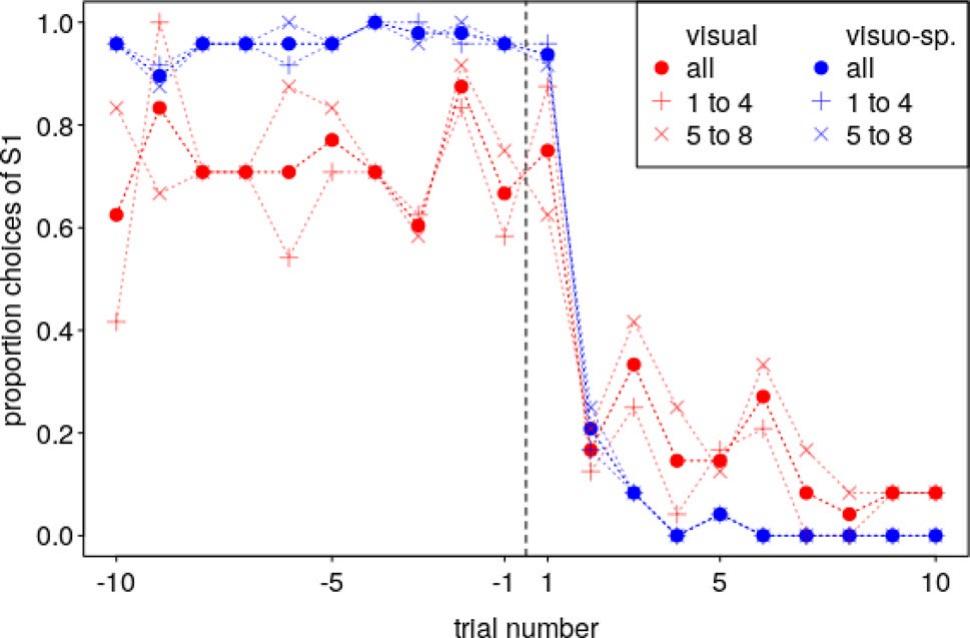
Proportion trials in which the birds chose the first correct stimulus (S1) in the shifted reversal experiment. Note that this proportion was still high in the first trial after the reversal (vertical dashed line) but then steeply decreased. Note also that the visuo-spatial group performed better than the visual group. Negative trial numbers indicate trials before the reversal

Across all sessions, in the midsession reversal experiment, the visuo-spatial group showed about 90% correct choices before and after the reversal, whereby they made particularly many errors in the first few trials after the reversal (Fig. 2). The visual group committed more errors and showed about 70% correct trials before and 70–90% correct trials (decreasing with increasing trial number) after the reversal. Also, the birds of this group made particularly many errors in the first few trials after the reversal. Both groups performed more accurately in the last as compared to the first ten sessions. The overall performance was not much different in the shifted reversal as compared to the midsession reversal experiment, and kea even seemed to commit fewer errors in the shifted reversal experiment (Fig. 3).

### Models

#### Midsession reversal, anticipatory errors (model 1)

**Fig. 4:**
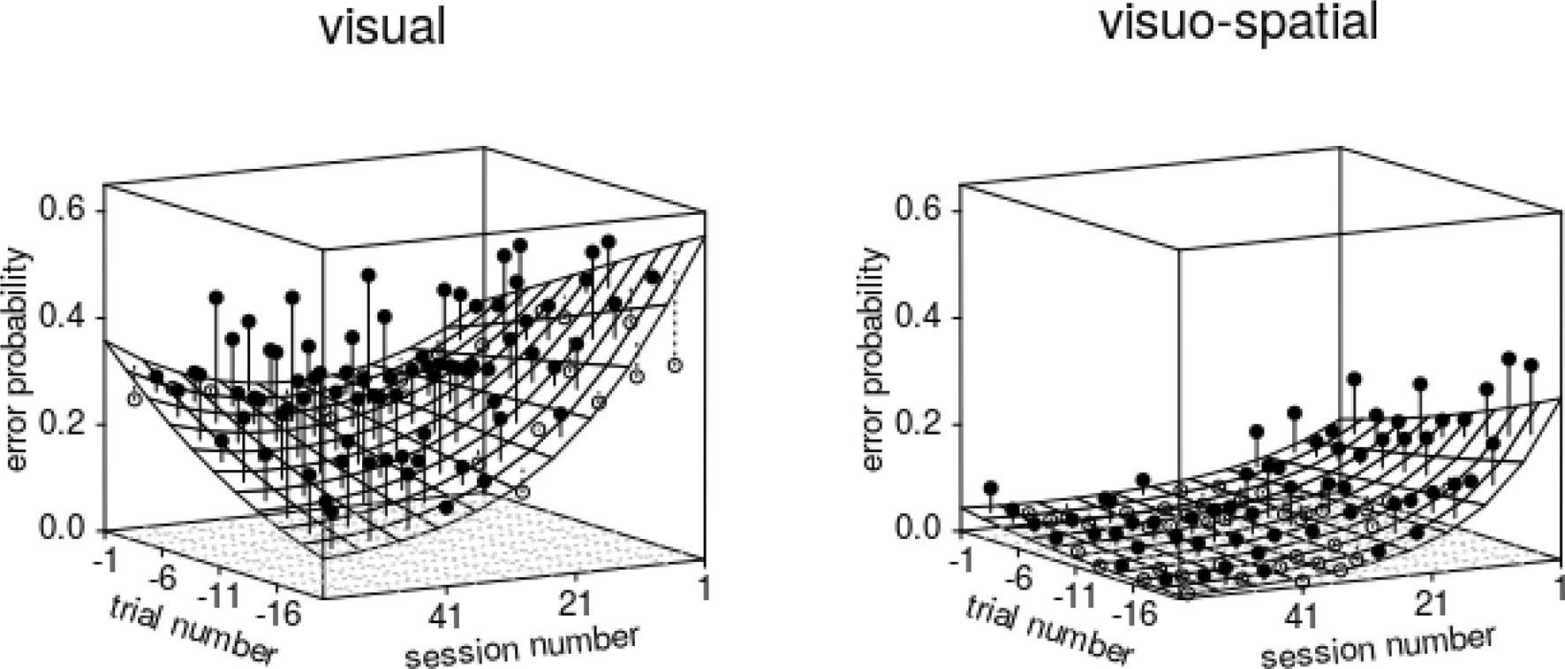
Probability of anticipatory errors in midsession reversal trials. Depicted are the fitted model (with *sex* manually dummy coded and then centred; surface) as a function of *trial number* until the reversal (− 1 corresponds to the trial immediately before the reversal), *session number*, and *group*. The dots show the average probability of an error per cell of the surface, whereby filled dots depict probabilities larger and open dots probabilities smaller than the fitted model. Note that the graph’s orientation was chosen for best visibility of the model plane, because of which trial and session numbers increase from right to left. The plots' back left edges correspond to the reversal’s position

The predictors *trial number*, *session number*, *group* and/or their interactions had a clear effect on the outcome variable *error probability*, as demonstrated by the full-null model comparison, which was clearly significant (likelihood ratio test: χ^2^ = 29.049, *df* = 11, *P* = 0.002). However, the three-way interaction between *session number* squared, *trial number*, and *group* appeared non-significant (Table SI 1). After removing this and other non-significant interactions (see Tables SI 1–SI 4), we found a clearly significant interaction between *session number* and *trial number* and a marginally non-significant interaction between *session number* and *group* (Table SI 4). More precisely, this means that birds in the visual group made a considerable proportion of errors in the beginning of the experiment, which slightly decreased over the course of the trials. At the end of the experiment they made few errors at the beginning of a session, but this probability then increased over the course of trials within sessions (Fig. 4). Birds in the visuo-spatial group overall made fewer errors than those in the visual group, and this probability did not vary much over the course of trials within sessions but clearly decreased over the course of the experiment.

#### Midsession reversal, perseverative errors (model 2)

**Fig. 5:**
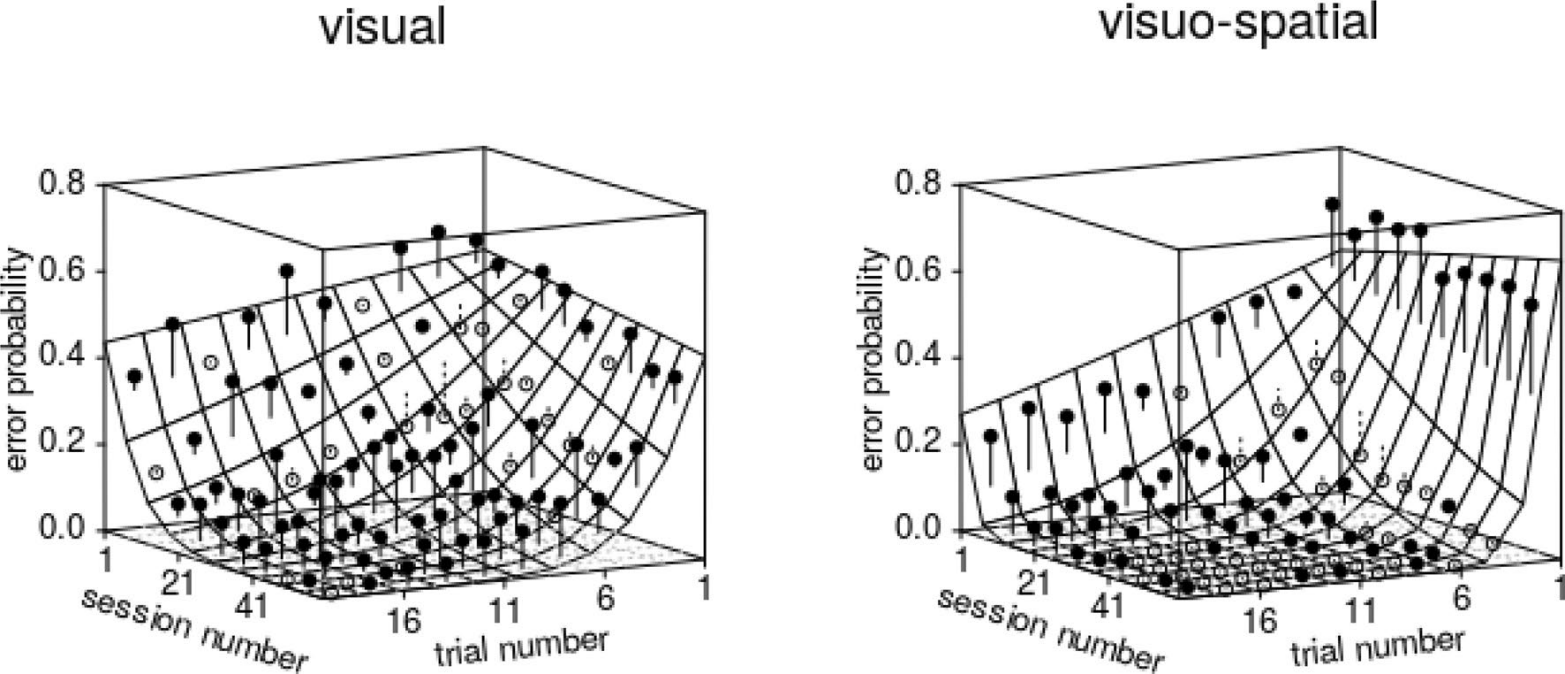
Probability of perseverative errors in midsession reversal trials. Depicted are the fitted model (with *sex* manually dummy coded and then centred; surface) as a function of *trial number* since the reversal (1 corresponds to the trial immediately after the reversal), *session number*, and *group*. The dots show the average probability of an error per cell of the surface, whereby filled dots depict probabilities larger and open dots probabilities smaller than the fitted model. Note that axes for *trial number* and *session number* are opposite to Fig. 4 and that *trial number* increases from right to left. The plots' right back edges correspond to the reversal’s position

Overall, the test predictors as a collective had a clear impact on the probability of perseverative errors (full-null model comparison: χ^2^ = 29.135, *df* = 7, *P* < 0.001), and there was a clearly significant three-way interaction between *trial number*, *session number*, and *group* (Table SI 5). More specifically, the probability of perseverative errors was high immediately after the reversal, decreased over the course of trials within sessions, and this decrease got steeper over the course of sessions, whereby this effect was much more pronounced in the visuo-spatial group (Fig. 5).

#### Shifted reversal, anticipatory errors (model 3)

**Fig. 6:**
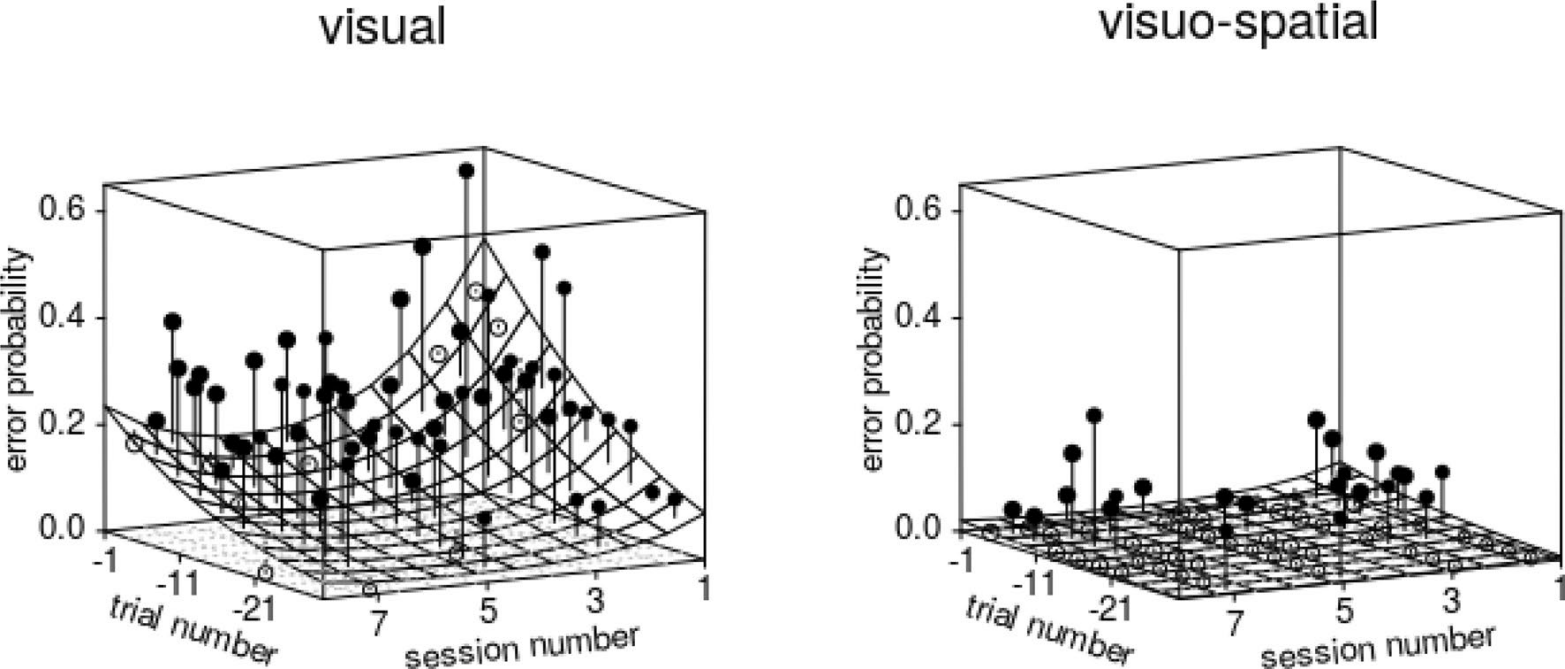
Probability of anticipatory errors in shifted reversal trials. Depicted are the fitted model (with *sex* manually dummy coded and then centred; surface) as a function of *trial number* until the reversal (− 1 corresponds to the trial immediately before the reversal), *session number*, and *group*. The dots show the average probability of an error per cell of the surface, whereby filled dots depict probabilities larger and open dots probabilities smaller than the fitted model. The 'volume' of the points corresponds to the number of trials in the respective cell of the surface (*N* = 9–18). Note that axes for *trial number* and *session number* are comparable to Fig. 4 and that trial and session numbers increase from right to left. The plots' back left edges correspond to the reversal’s position

Also in the shifted reversal experiment, the probability of anticipatory errors was clearly influenced by the collective of the test predictors (χ^2^ = 28.119, *df* = 11, *P* = 0.003), and again, as in the midsession reversal experiment, the three-way interaction between *trial number*, *session*, and *group* appeared non-significant (Table SI 6). After removal of this and all other interactions, which all turned out being non-significant (Tables SI 6–SI 8), we found clear effects of *trial number*, *session* (linear and squared), and *group* (Table SI 9). The probability of errors was much higher in the visual group, increased over the course of trials within sessions, whereby this effect was more pronounced in the early and late sessions (Fig. 6).

#### Shifted reversal, perseverative errors (model 4)

**Fig. 7:**
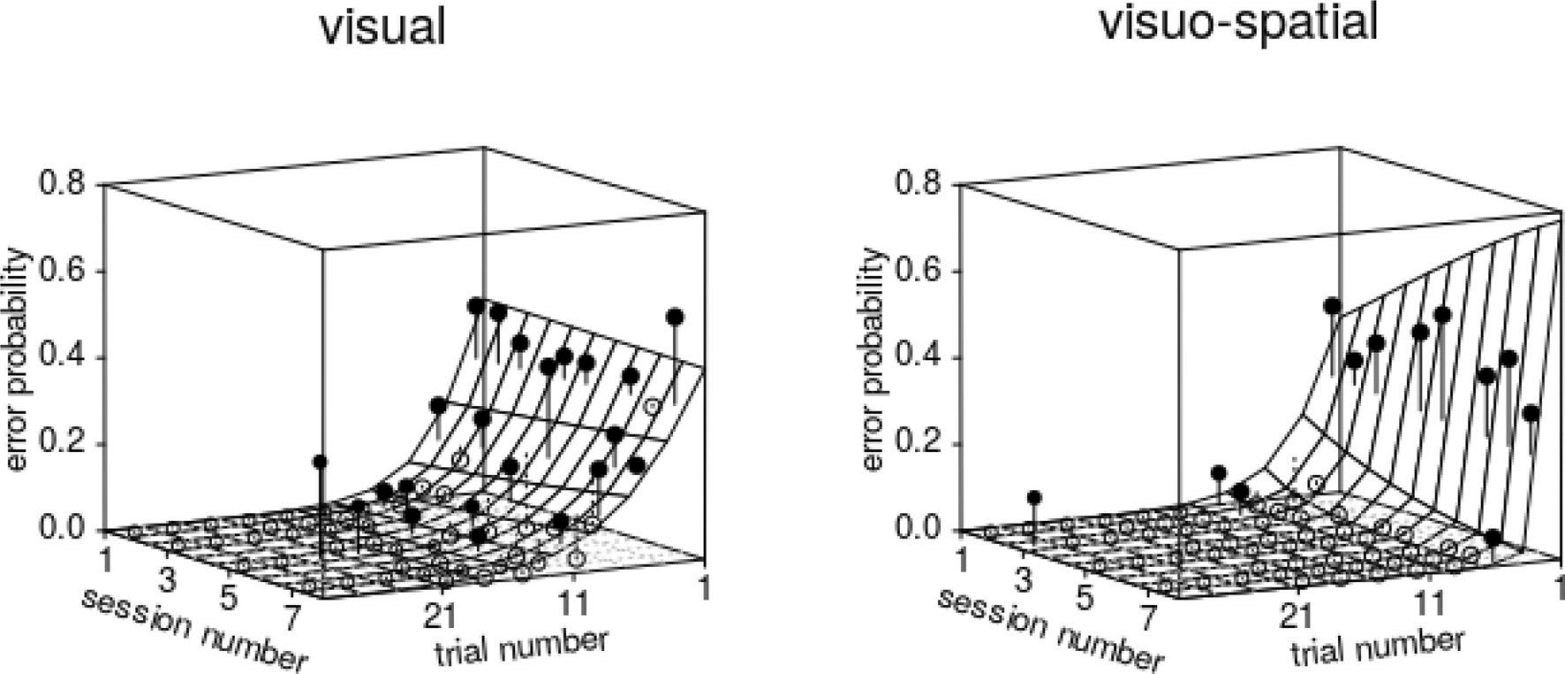
Probability of perseverative errors in shifted reversal trials. Depicted are the fitted model (with *sex* manually dummy coded and then centred; surface) as a function of *trial number* since the reversal (1 corresponds to the trial immediately after the reversal), *session number*, and *group*. The dots show the average probability of an error per combination of cell of the surface, whereby filled dots depict probabilities larger and open dots probabilities smaller than the fitted model. The 'volume' of the points corresponds to the number of trials in the respective cell of the surface (*N* = 9–18). Note that axes for *trial number* and *session number* are opposite to Fig. 6 and that *trial number* increases from right to left. The plots' right back edges correspond to the reversal’s position

Finally, also the probability of perseverative errors in the shifted reversal experiment was clearly influenced by the collective of the test predictors (χ^2^ = 76.900, *df* = 7, *P* < 0.001). As in the midsession reversal experiment, we found a clearly significant interaction between *trial number*, *session number*, and *group* (Table SI 10), whereby we found that the probability of errors decreased with trial number within sessions, that this effect got more pronounced over the course of sessions and particularly so in the visuo-spatial group (Fig. 7).

### Variation among birds and sessions

We found in part considerable variation among birds and also among sessions within birds as indicated by the estimated contributions of the random effects *individual* and *session ID* nested within *individual*. Many of the estimated standard deviations were in the same order of magnitude as the respective fixed effect (compare Tables SI 11–14 with Tables SI 1, 5, 6, and 10). For instance, in the model of perseverative errors after the midsession reversal (model 2) we found the fixed effect of *group* estimated as − 3.811 (Table SI 5) whereas the random intercept of *individual* was estimated to contribute with a standard deviation of 2.215 (Table SI 12), which indicates that the variation among birds was considerable, when compared to the difference between groups (Fig. SI 3). Similarly, the fixed effect of *trial number* (at session being at its average and for the visual group) was estimated to be -1.610 (Table SI 5) whereas the random slope of trial number within *individual* was estimated to contribute with a standard deviation of 1.537 (Table SI 12), indicating that the effect of trial number varied considerably between birds. Indeed, there was considerable variation among birds with regard to their overall probability of committing errors, particularly in the visual group (Fig. SI 3–7). Furthermore, the session to session variation in the probability to commit errors varied in part considerably within birds, and also the effect of *trial number* varied in part considerably among and within birds. Examples of the variability, but also accuracy, in the kea’s responses are provided in the supporting information (SI_1) as figures for the last five sessions of the midsession reversal task (Fig. SI 4 and 5) and all sessions of the shifted reversal task (Fig. SI 6 and 7) as well as with figures for every individuals’ performance in every even session of the midsession reversal task (Fig. SI 8–19).

An analysis for specific response patterns revealed that six birds of both groups had produced at least one completely correct session (with 40 trials out of 40 correct), and birds from both groups, but especially from the visuo-spatial group, showed perfect or near perfect win-stay/lose-shift sessions in both tasks (Table 2), meaning they had made only one error at trial 21 or two errors at trial 21 and trial 22 but were otherwise always correct.

**Table 2:** Characteristics of individual performance

		nr. sessions no error^a^		only 1 at 1^b^		only 2 at 1 and 2^c^	
Group	Individual	MSR	SR	MSR	SR	MSR	SR
Visual	Coco	0	0	0	0	0	0
Visual	John	3	0	4	0	0	1
Visual	Papu	0	0	0	0	0	0
Visual	Pick	0	0	0	0	0	0
Visual	Roku	0	0	9	4	3	0
Visual	Willy	1	0	8	4	4	0
Visuo-sp.	Anu	1	0	10	2	0	1
Visuo-sp.	Frowin	1	0	23	6	3	0
Visuo-sp.	Lilly	0	0	9	3	16	3
Visuo-sp.	Mali	0	0	6	3	11	1
Visuo-sp.	Paul	0	1	20	6	3	1
Visuo-sp.	Plume	0	1	12	4	1	1

MSR denotes the midsession reversal experiment and SR the shifted reversal experiment

The total number of sessions were 60 (MSR) and 8 (SR)

^a^Number of sessions with no error at all

^b^Number of sessions in which birds committed only one error and this occurred at the first trial after the reversal

^c^Number of sessions in which birds committed only two errors and these occurred at the first and second trial after the reversal

## Discussion

With this study we are able to provide strong evidence for the kea’s ability to quickly and successfully adapt to the change of reinforcement contingencies in a midsession reversal and a shifted reversal test. It rests on our analytical approach with Generalized Linear Mixed Models (GLMMs) to model the probability of anticipatory and perseverative errors for the response patterns win-stay/lose-shift and reversal estimation as well as to estimate between- and within-individual variation in the responses to the reversal. Overall, our results suggest that the kea, on average, showed a response pattern more similar to win-stay/lose-shift than to reversal estimation, with nine out of twelve birds even showing perfect or near perfect win-stay/lose-shift choice patterns in at least one session. There was an effect of task requirement, with this pattern being more frequently shown in the visuo-spatial group as compared to the visual group, suggesting an adoption of win-stay/lose-shift as a general strategy within the visuo-spatial group. Nonetheless, considerable individual differences were noticeable between and within birds.

Kea of both groups (visual and visuo-spatial) learned to adjust their choice behaviour to the midsession reversal paradigm and improved their success over time. Especially birds of the visuo-spatial group showed a high initial performance. In line with our hypothesised error probabilities, we found clear evidence for win-stay/lose-shift in the visuo-spatial group. The models indicated that the probability of errors did not increase towards the reversal but was high immediately after the reversal with a rapid decrease thereafter as hypothesised (Fig. 1i, l). This result fits into the results provided for humans (Cook and Rosen 2010; Rayburn-Reeves et al. 2011), rats (Rayburn-Reeves et al. 2013b), and macaques (Rayburn-Reeves et al. 2017). We had also hypothesised that for the case of the adoption of reversal estimation, errors should gradually become more likely before and less likely after the reversal (Fig. 1b, e) and as proficiency in estimating the reversal is reached, no errors should occur anymore (Fig. 1c, f). This proficiency was not found as a stable response pattern over time, but only in single sessions by single individuals. Two of the six birds of the visual group adopted neither win-stay/lose-shift nor reversal estimation but showed a side bias in the first half of the session and then followed the correct stimulus in the second half with comparable accuracy to their group members. This side bias decreased the group performance in the first half and likely had an effect on models 1 and 3, as in the visual group the correct stimulus was presented an equal number of times on either side and a side bias lead to a maximum of only 50% correct in the first half of the session. The heterogeneity of the visual group hence implies that considering only group performance (i.e., an average across birds) can be misleading and might blur between individual variation.

In contrast to the visuo-spatial group, in the midsession reversal experiment, the visual group’s probability of anticipatory errors increased with trial number towards the reversal. In the beginning of the experiment the probability of perseverative errors needed more trials to decrease, which hints at birds estimating when the reversal will take place instead of relying on the local information of reinforcement. However, in the same group we also found that the probability of perseverative errors in midsession reversal trials was high in the first trials after the reversal but then clearly decreased with trial number, whereby this effect became much stronger over the course of sessions, which means that the later the session, the more the kea of the visual group also relied on the local information of reinforcement. This finding, together with the fact that the probability of anticipatory errors did not change much over the course of the trials prior to the reversal (Fig. 2), might suggest that individuals of the visual group were in part also adopting a win-stay/lose-shift strategy, even if the overall accuracy of the group was less than optimal (compare Fig. 1) due to the variation within and between birds. Overall, this complex pattern of choice is comparable to responses of humans (Rayburn-Reeves et al. 2011 sessions 6–10) and rats (Smith et al. 2016), but contrasts with pigeons (e.g. Cook and Rosen 2010; Rayburn-Reeves et al. 2011, 2013b; Rayburn-Reeves and Cook 2016) and dogs (Laude et al. 2016) that mainly showed a gradual decrease of responses to the first correct stimulus when estimating the reversal. However, we detected in some pigeon studies (see Stagner et al. 2013 time out group; Laude et al. 2014, 2016; McMillan et al. 2014 spatial transfer group) a comparable drop in responses to the first correct stimulus after the reversal, although this was not discussed in detail by the authors. As the kea’s individual performance changed from session to session between the responses win-stay/lose-shift and reversal estimation, it would be interesting to know whether individuals of other species change their responses between sessions as well. To answer this important question, it would be necessary to consider individual differences and also within-individual differences between sessions in the analysis.

Our models revealed a clear difference in performance between the visual and visuo-spatial group, suggesting an effect of task requirement. Both groups of kea made their stimulus choices uninterruptedly and quickly which could lead to mistakes, but we assume that the birds of the visual group were more prone to errors because they were additionally required to flexibly shift their attention to follow the correct stimulus, which changed the position in an unpredictable way. Spatial information as an additional discriminating cue simplified the task, and the spatial consistency of the stimuli allowed for more optimal choices, leading to more win-stay/lose-shift sessions. We cannot exclude for the visuo-spatial group that the use of body orientation had an influence on adopting win-stay/lose-shift (see Rayburn-Reeves et al. 2013a; McMillan et al. 2014). However, individuals from the visual group showed win-stay/lose-shift patterns as well, without being able to use their body orientation. This suggests that spatial information may be less important for kea than for pigeons to adopt win-stay/lose-shift (Rayburn-Reeves et al. 2013a; Laude et al. 2014; McMillan et al. 2014), although choice patterns from individual pigeon sessions would be needed to confirm this.

The models for the shifted reversal task demonstrated that the visual group showed more anticipation attempts of the now unpredictable reversal than the visuo-spatial group. However, the birds of the visual group also reacted more sensitively to the reversal information in the shifted reversal task than in the midsession reversal task, meaning that they used the local information of reinforcement more frequently. Birds from both groups were not able to increase their number of sessions with a perfect win-stay/lose-shift, which would be the only reliable way to maximise their rewards in a shifted reversal task. As we tested only eight sessions per bird, results need to be considered with caution. It seems likely that at least some of the kea explored the new contingencies instead of following what they had learned in the midsession reversal task. This is consistent with our observation that error rates did not necessarily increase at the trials around the new reversals but at trials in other parts of the sessions, or that specific response patterns, like a repeated left–right side shift, occurred in some birds.

While, on average, our study birds adopted win-stay/lose-shift, we also found in part considerable variation between individuals and also within individuals between sessions independently from their test group. Birds from both groups managed to achieve exact win-stay/lose-shift sessions (see Fig. SI 4 and 5) with only one or two errors immediately after the reversal, which is comparable to humans adopting win-stay/lose-shift (Cook and Rosen 2010; Rayburn-Reeves et al. 2011). It seemed that win-stay/lose-shift was not a mere result of a reward following repetitive behaviour, but early sessions suggest that the birds needed to acquire this response to the reversal. Also, birds from both groups showed the ability of an accurate reversal estimation, which is incompatible with a win-stay/lose-shift strategy by definition. Although showing a preference for one type of response to the reversal (mainly win-stay/lose-shift), nine out of twelve birds showed both patterns in the course of the experiment.

Previous studies relied on pooled data and reported individual performances mainly to demonstrate that the pooled data of single individuals matched the pooled data of the group (Cook and Rosen 2010; Rayburn-Reeves et al. 2011, 2017; McMillan et al. 2016). Only two studies mentioned variability between individuals (McMillan et al., 2014; McMillan and Roberts, 2015), but the authors did not report data. Responses of rats (Smith et al. 2016) and two individual pigeons (Rayburn-Reeves and Cook 2016) that resemble the response patterns of our kea raise the question whether the use of both strategies is only undetected in other species. We encourage the reader to explore the individual kea’s responses in the supporting information (Fig. SI 8–19) and suggest that future studies in this field should also analyse between- and within-individual variation in performance and possible causes of such variation.

We can only speculate why the two birds of the visual group showed a side bias in their first half of the sessions. Both birds were fully motivated to participate, nevertheless with the side bias they were able to achieve a maximum of 30 correct trials, assumedly a fairly good outcome for the fully fed birds. During the entire test they never learned their first correct stimulus, although their responses suggest that they reacted to the reversal information and their second correct stimulus. Six out of twelve birds learned their second correct stimulus first, which seemed counterintuitive, but it can be assumed that the second correct stimulus is easier to learn as it is a more valid and reliable predictor for the choices’ outcome as the second correct stimulus never reverses within a session (Rayburn-Reeves and Cook 2016). Additionally, it seems possible, that they focussed on only one stimulus (the second correct) during the whole test, instead of both stimuli according to their contingency of reinforcement in each half of the test. Although capable of inference by exclusion, kea were also shown to not always rely on this ability (O’Hara et al. 2016). A comparable formation of independent rules for each stimulus was suggested for pigeons in a go/no-go midsession reversal task by McMillan and colleagues (2015).

Without further tests we cannot yet draw firm conclusions about the various factors that could influence the performance and individual variation in a midsession reversal learning test. Like in other species it is known that the capabilities, personality, and individual motivations of psittacines contribute to their individual differences (Cussen 2017). Additionally, as we tested outdoors, the kea were exposed to sounds of other birds, or sometimes animal keepers passing by, as well as environmental factors like wind during testing. This might influence some birds more than others in their level of attention to the test. Future tests will need to address the underlying mechanisms that enable some birds to estimate the reversal with high accuracy and why they changed their response strategy to do so even when local information was given.

In general, it seems that factors like impulse control, attention, memory, the ability to estimate time intervals, as well as the ability to recognize sequences or patterns over time, can influence the response in a midsession reversal learning task. Without further tests, we can only speculate about the mechanisms behind our kea’s responses. As most kea were able to change response strategies between sessions, it seems unlikely that in kea anticipatory and perseverative errors are predominantly caused by global cues overruling local cues as has been suggested for pigeons (Rayburn-Reeves and Cook 2016). Nevertheless, to draw firm conclusions, these mechanisms and whether they are influenced by the time into the session or trial number need further testing in kea, for example in setups with different intertrial-intervals. Additionally, we found the responses in sessions with a correctly estimated reversal to be very rhythmical. Further studies therefore should investigate whether rhythmic behaviour could have helped the birds to keep track of the reversal. Furthermore, to adopt win-stay/lose-shift and therefore prevent anticipatory and perseverative errors, it seems crucial to have the impulse control to wait for the information of the first trial after the reversal. Kea waited up to 160 s in a food exchange task (Schwing et al. 2017), and the ability to do so could be an advantage when adopting win-stay/lose-shift. Pigeons and dogs, who performed suboptimally in most midsession reversal studies (e.g. Cook and Rosen 2010; Laude et al. 2016) also experienced difficulties when tested for impulsive behaviour. Pigeons performed poorly in impulse control studies (Ainslie 1974), and whether dogs are able to control themselves depends on the way it is tested (Marshall-Pescini et al. 2015; Brucks et al. 2017). Kea are known for their curious and explorative behaviour (Diamond and Bond 1999; O’Hara et al. 2012), and previous studies on kea have reported the importance of trial and error learning and exploration for their problem-solving abilities (Miyata et al. 2011; Gajdon et al. 2013; Lambert et al. 2017). Exploring new possible responses has also led to dismissing already learned solutions (Gajdon et al. 2011) or led to inducing unrewarded responses for a while, possibly to assess the consequences (Liedtke et al. 2011; Stobbe et al. 2012). In a test of insightful problem solving—string pulling—kea continued to try out alternative solutions despite being very successful with the typical beak-foot coordination before (Werdenich and Huber 2006).

In conclusion, with this study we could show that an avian species is able to exhibit efficient response strategies in the midsession reversal task. Kea parrots have shown flexibility and variability in their test performance by showing different response patterns. On the one hand they exploited local information of reinforcement, i.e., the outcome of the previous trial, to quickly adapt to the task affordances, which has proven particularly effective in sessions with unpredictable reversals. On the other hand, they sometimes solved the task with a notable high accuracy by estimating the reversal. Knowing the explorative and neophilic nature of the kea’s foraging behaviour we have not been surprised to find considerable inter- and intra-individual variation. Still, the occurrence of completely correct sessions requires a second look into their response behaviour, taking into account the possibility that the kea used internal cues, like timing, to accurately estimate the reversal, as a strategy to be employed conjointly or alternatively to win-stay/lose-shift. In addition to physical tasks, in which kea have already proven their flexibility and insightfulness, up to inventing tool use, the midsession reversal task seems to us a perfect paradigm to investigate the mind of the kea.

## Supplementary Information

Below is the link to the electronic supplementary material.

Supplementary file1 (PDF 2627 kb)

Supplementary file2 (zip 209 kb)

Supplementary file3 (XLSX 1041 kb)

## Data Availability

The datasets generated and analysed during the current study are available in the supporting information (SI_2 and SI_3).

## Code availability

The code generated during the current study is available in the supporting information (SI_2).
