# Supplementary material for: Kea (Nestor notabilis) show flexibility and individuality in within-session reversal learning tasks
Source: Anim Cogn. 2021 Jun 10;24(6):1339–51. doi: 10.1007/s10071-021-01524-1 (PMC8492579; doi:10.1007/s10071-021-01524-1)
Supplement: Supplementary file 1 — Supplementary file1 (PDF 2627 kb) [file 10071_2021_1524_MOESM1_ESM.pdf]

Supporting information

Kea (*Nestor notabilis*) show flexibility and individuality in within-session reversal learning tasks

Animal Cognition, Springer

Monika Laschober [1], Roger Mundry[1, 2], Raoul Schwing[1], Ludwig Huber[1]

[1] Comparative Cognition, Messerli Research Institute, University of Veterinary Medicine Vienna, Medical University of Vienna, University of Vienna, Vienna, Austria

[2] Platform Bioinformatics and Biostatistics, University of Veterinary Medicine Vienna, Vienna, Austria

Corresponding author: Monika Laschober; monika.laschober@gmx.at

Content

Statistical analysis 2

Initial and final full models fitted 4

Detailed model results 6

Additional Figures 14

References 33

Dear reader,

this supporting information provides a full description of the statistical analysis, including model formation decisions, as well as the initial and final models for all four models of the paper. It includes detailed model results for the fixed and random effects parts of the models (Table SI 1 to 14). Additionally, we provide figures to illustrate how to interpret the relative contribution of a random intercept or a random slope (Fig. SI 1 and 2), a figure of the probability of perseverative errors (Fig. SI 3), figures of the probability and occurrence of anticipatory or perseverative errors per bird in the last five sessions of the midsession reversal task (Fig. SI 4 and 5) and the eight sessions of the shifted reversal task (Fig. SI 6 and 7). Following that, you will find figures illustrating the individuals' performance in every even session of the midsession reversal task (Fig. SI 8 to 19) to illustrate the variety, but also the accuracy of responses shown by the kea. Finally, we provide an overview of the proportion choices of S1 per bird (Fig.SI 20) to simplify the comparison with previous studies.

Statistical analysis

In order to model the probability of anticipatory and perseverative errors we fitted a total of four Generalized Linear Mixed Models (GLMM; Baayen, 2008), one for each combination of the type of error (anticipatory and perseverative) and reversal timing (midsession or shifted reversal). The four models hence differed in the data used, whereby for modelling the probability of anticipatory errors (models 1 and 3) we used only the trials before the reversal and for modelling the probability of perseverative errors (models 2 and 4) we used only the trials after the reversal. The fitted models, however, were largely identical for each of the four subsets. The response was the occurrence of an error at the individual trial, and hence we fitted the model with binomial error structure and logit link function (McCullagh and Nelder 1989). Into the models of anticipatory errors, we included as key fixed effects the trial number, session number (linear and squared), and group (visual or visuo-spatial), and all their interactions up to the third order. Into the models of perseverative errors, we included trial number, session number, and group, and all their interactions up to the third order as the key fixed effects. The reason why we included session number squared and its interactions with group and trial number into models of anticipatory but not those of perseverative errors was that we had a respective prediction regarding anticipatory but not perseverative errors (see Fig. 1). To control for their effects, we also included age and sex as further fixed effects in all models. To model variation among individuals and from session to session and to avoid pseudo-replication we included random intercept effects of individual and session ID nested within individual. To keep type I error rate at the nominal level of 0.05 and in order to being able to infer about strategies varying between individuals and/or from session to session within individuals, we included random slopes (Schielzeth and Forstmeier 2008; Barr et al. 2013) of trial number, session number (linear and squared, if present in the model), and their interactions within individual and of trial number within session ID. We initially also included the correlations among random intercepts and slopes, but in case they appeared in part unidentifiable (as indicated by many absolute correlation parameters estimated as essentially 1; Matuschek, Kliegl, Vasishth, Baayen, & Bates, 2017), we removed them from the model (see the supporting information for the final full models fitted). To avoid 'cryptic multiple testing' (Forstmeier and Schielzeth 2011) we compared each full model with a respective null model lacking trial number, session number, group, and all their interactions in the fixed effects part but being otherwise identical.

For the purpose of exploring the magnitude of variation among and within birds, we first compared the standard deviation estimated for the contribution of the random effects with the value of the respective fixed effect. Such a comparison is possible since both indicate the influence of the respective effect on the response on the same scale (“link space”; McCullagh and Nelder 1989). For instance, if the fixed effect of group would be estimated to have a value of 1 and the random intercept of individual would be estimated to have a value of 1, too, then the variation among individuals would be of considerable magnitude as compared to the difference between groups, and, in link space, individuals from the same group could differ more from one another than do the averages of the two groups (Fig. SI 1). Similarly, if the fixed effect of trial number (given a certain session and group) would be estimated to be 1, and the random slope of trial number within individual would also be estimated to be 1, then the variation among individuals with regard to the effect of trial number would be large as compared to the overall effect of trial number (Fig. SI 2). Second, we extracted Best Linear Unbiased Predictors (BLUPs; Baayen

2008) and plotted the estimated individual specific effects together with group level effects and the individual observations to get an impression about the magnitude of variation among and between individuals.

We fitted the models in R (version 3.6.3; R Core Team 2020) using the function glmer of the package lme4 (version 1.1-21; Bates et al. 2015) utilizing the optimizer 'bobyqa'. Prior to fitting the models, we z-transformed trial number, session number, and age to ease model convergence and achieve easier interpretable model coefficients (Schielzeth 2010). We conducted full-null model comparisons by means of likelihood ratio tests (Dobson 2002) and tested individual fixed effects by dropping them from models one at a time and utilizing a likelihood ratio test to compare the simpler with the more complex model (Barr et al. 2013). In case a full-null model comparison revealed significance, but the highest order interactions did not, we iteratively removed non-significant interactions (beginning with the highest order interactions) until none of the non-significant interactions were left. We did so in order to be able to infer about the respective lower order terms (we report the full results for all models in the supporting information). We estimated the stability of the full models by excluding the levels of the random effects one at a time, fitted the full model to each of these subsets, and compared the estimates obtained for the subsets with those obtained for the respective full data set. This revealed the models to be of moderate to good stability (see results). We determined confidence intervals of the model estimates and fitted values by means of a parametric bootstrap (N=1000 bootstraps; function bootMer of the package lme4). In case of the shifted reversal experiments (models 3 and 4) we aligned trial number according to when the reversal occurred; that is, in all sessions the last trial before the reversal got the same trial number, and the first trial after the reversal got the same trial number, too. The samples analysed for these models comprised 14400 trials conducted in 720 sessions with 12 birds (midsession reversal, model of anticipatory as well as perseverative errors) or 1920 trials conducted in 96 sessions with 12 birds (shifted reversal, model of anticipatory as well as perseverative errors), respectively. All Figures were created in R (version 3.6.3; R Core Team 2020).

Initial and final full models fitted

Midsession reversal, anticipatory errors (model 1)

Initial model:

glmer(error~z.trial.nr*(z.Session+I(z.Session^2))*Group+z.Age+Sex+

(1+z.trial.nr*(z.Session+I(z.Session^2))|Bird_name)+(1+z.trial.nr|sessionID),

family=binomial, data=msr.pre.data)

Final model:

glmer(error~z.trial.nr*(z.Session+I(z.Session^2))*Group+z.Age+Sex+

(1+z.trial.nr*(z.Session+I(z.Session^2))|Bird_name)+(1+z.trial.nr||sessionID),

family=binomial, data=msr.pre.data)

The log-likelihoods of the models were -5331.244 (initial) and -5338.604 (final).

Midsession reversal, perseverative errors (model 2)

Initial model:

glmer(error~z.trial.nr*z.Session*Group+z.Age+Sex+

(1+z.trial.nr*z.Session|Bird_name)+(1+z.trial.nr|sessionID),

family=binomial, data=msr.post.data)

Final model:

glmer(error~z.trial.nr*z.Session*Group+z.Age+Sex+

(1+z.trial.nr*z.Session|Bird_name)+(1+z.trial.nr||sessionID),

family=binomial, data=msr.post.data, control=contr)

full.msr.post.wnc=glmer(error~z.trial.nr*z.Session*Group+z.Age+Sex+

(1+z.trial.nr*z.Session||Bird_name)+(1+z.trial.nr||sessionID),

family=binomial, data=msr.post.data)

The log-likelihoods of the models were -4631.652 (initial) and -4673.866 (final).

Shifted reversal, anticipatory errors (model 3)

Initial model:

glmer(error~z.trial.nr*(z.Session+I(z.Session^2))*Group+z.Age+Sex+

(1+z.trial.nr*(z.Session+I(z.Session^2))|Bird_name)+(1+z.trial.nr|sessionID),

family=binomial, data=sr.pre.data)

Final model:

glmer(error~z.trial.nr*(z.Session+I(z.Session^2))*Group+z.Age+Sex+

(1+z.trial.nr*(z.Session+I(z.Session^2))|Bird_name)+(1+z.trial.nr||sessionID),

family=binomial, data=sr.pre.data)

The log-likelihoods of the models were -529.516 (initial) and -532.404 (final).

Shifted reversal, perseverative errors (model 4)

Initial model:

glmer(error~z.trial.nr*z.Session*Group+z.Age+Sex+

(1+z.trial.nr*z.Session|Bird_name)+(1+z.trial.nr|sessionID),

family=binomial, data=sr.post.data)

Final model:

glmer(error~z.trial.nr*z.Session*Group+z.Age+Sex+

(1+z.trial.nr*z.Session||Bird_name)+(1+z.trial.nr||sessionID),

family=binomial, data=sr.post.data)

The log-likelihoods of the models were -344.608 (initial) and -366.268 (final).

Detailed model results

Table SI 1 Results of the fixed effects part of the full model of anticipatory errors in the midsession reversal experiment (model 1; estimates together with confidence limits, significance tests, and range of estimates obtained when dropping levels of random effects one at a time).

term[1]	Estimate	SE	lower cl	upper cl	c2	df	P	min	max
intercept	-1.322	0.440	-2.209	-0.457			[2]	-1.635	-0.608
trial nr.	0.168	0.106	-0.028	0.379			[2]	0.077	0.243
session nr.	-0.403	0.144	-0.686	-0.126			[2]	-0.542	-0.305
session nr.^2	0.157	0.089	-0.020	0.326			[2]	0.096	0.211
group	-2.167	0.555	-3.181	-1.158			[2]	-2.572	-1.611
z.Age	-0.231	0.241	-0.776	0.296	0.874	1	0.350	-0.458	0.447
SexM	0.157	0.507	-0.849	1.149	0.092	1	0.761	-1.168	0.856
trial nr.:session nr.	0.212	0.083	0.056	0.380			[2]	0.136	0.264
trial nr.:session nr.^2	-0.014	0.067	-0.139	0.124			[2]	-0.057	0.039
trial nr.:group	-0.013	0.163	-0.328	0.305			[2]	-0.093	0.104
session nr.:group	-0.423	0.211	-0.865	0.012			[2]	-0.522	-0.297
session nr.^2:group	0.072	0.136	-0.182	0.344			[2]	-0.018	0.153
trial nr.:session nr.:group	0.083	0.128	-0.184	0.322	0.391	1	0.532	-0.055	0.156
trial nr.:session nr.^2:group	-0.016	0.106	-0.232	0.185	0.020	1	0.886	-0.119	0.046

[1] trial number and session number were z-transformed to a mean of zero and a standard deviation of one, mean (sd) of the original variable were 10.5 (5.766) and 30.5 (17.319), respectively; group was dummy coded with visual=0 and visuo-spatial=1

[2] not indicated because of having a very limited interpretation

Table SI 2 Results of the fixed effects part of the model of anticipatory errors in the midsession reversal experiment (model 1) after removal the non-significant highest order interactions (estimates together with confidence limits and significance tests).

term[1]	Estimate	SE	lower cl	upper cl	c2	df	P
intercept	-1.333	0.434	-2.144	-0.546			[2]
trial nr.	0.174	0.096	-0.008	0.365			[2]
session nr.	-0.403	0.144	-0.706	-0.113			[2]
session nr.^2	0.159	0.089	-0.015	0.333			[2]
group	-2.145	0.532	-3.256	-1.069			[2]
z.Age	-0.231	0.241	-0.745	0.281	0.877	1	0.349
SexM	0.156	0.507	-0.783	1.191	0.091	1	0.763
trial nr.:session nr.	0.209	0.080	0.054	0.368			[2]
trial nr.:session nr.^2	-0.020	0.052	-0.121	0.086	0.135	1	0.713
trial nr.:group	-0.029	0.121	-0.266	0.238			[2]
session nr.:group	-0.424	0.211	-0.896	0.024			[2]
session nr.^2:group	0.069	0.135	-0.227	0.328	0.240	1	0.624
trial nr.:session nr.:group	0.091	0.115	-0.144	0.323	0.587	1	0.444

[1] trial number and session number were z-transformed to a mean of zero and a standard deviation of one, mean (sd) of the original variable were 10.5 (5.766) and 30.5 (17.319), respectively; group was dummy coded with visual=0 and visuo-spatial=1

[2] not indicated because of having a very limited interpretation

Table SI 3 Results of the fixed effects part of model of anticipatory errors in the midsession reversal experiment (model 1) after removal of non-significant intermediate order interactions (estimates together with confidence limits and significance tests).

term[1]	Estimate	SE	lower cl	upper cl	c2	df	P
intercept	-1.441	0.394	-2.231	-0.648			[2]
trial nr.	0.173	0.074	0.028	0.323			[2]
session nr.	-0.440	0.140	-0.726	-0.142			[2]
group	-1.852	0.391	-2.760	-0.991			[2]
session nr.^2	0.187	0.068	0.039	0.324	5.534	1	0.019
z.Age	-0.235	0.245	-0.775	0.305	0.873	1	0.350
SexM	0.143	0.516	-0.891	1.164	0.074	1	0.786
trial nr.:session nr.	0.260	0.059	0.134	0.384	12.411	1	0.000
trial nr.:group	-0.074	0.099	-0.293	0.139	0.527	1	0.468
session nr.:group	-0.354	0.191	-0.801	0.051	2.938	1	0.087

[1] trial number and session number were z-transformed to a mean of zero and a standard deviation of one, mean (sd) of the original variable were 10.5 (5.766) and 30.5 (17.319), respectively; group was dummy coded with visual=0 and visuo-spatial=1

[2] not indicated because of having a very limited interpretation

Table SI 4 Results of the fixed effects part of model of anticipatory errors in the midsession reversal experiment (model 1) after removal of all non-significant interactions (estimates together with confidence limits and significance tests).

term[1]	Estimate	SE	lower cl	upper cl	c2	df	P
intercept	-1.430	0.395	-2.223	-0.599			[2]
session nr.	-0.435	0.140	-0.724	-0.135	5.726	1	0.017
trial nr.	0.142	0.061	0.020	0.262			[2]
group	-1.887	0.406	-2.797	-0.937			[2]
session nr.^2	0.189	0.068	0.048	0.325			[2]
z.Age	-0.238	0.245	-0.762	0.266	0.899	1	0.343
SexM	0.145	0.521	-0.836	1.131	0.074	1	0.785
session nr.:trial nr.	0.260	0.059	0.130	0.385	12.471	1	0.000
session nr.:group	-0.365	0.194	-0.804	0.058	3.013	1	0.083

[1] trial number and session number were z-transformed to a mean of zero and a standard deviation of one, mean (sd) of the original variable were 10.5 (5.766) and 30.5 (17.319), respectively; group was dummy coded with visual=0 and visuo-spatial=1

[2] not indicated because of having a very limited interpretation

Table SI 5 Results of the fixed effects part of the full model of perseverative errors in the midsession reversal experiment (model 2; estimates together with confidence limits, significance tests, and range of estimates obtained when dropping levels of random effects one at a time).

term[(1)]	Estimate	SE	lower cl	upper cl	c2	df	P	min	max
intercept	-2.403	0.915	-4.032	-0.668			[(2)]	-2.669	-1.847
trial nr.	-1.610	0.630	-2.733	-0.397			[(2)]	-1.925	-1.252
session nr.	-1.518	0.518	-2.463	-0.508			[(2)]	-1.772	-1.154
group	-3.811	1.306	-6.239	-1.267			[(2)]	-4.717	-2.922
z.Age	-0.020	0.136	-0.303	0.272	0.021	1	0.884	-0.196	0.279
SexM	-0.369	0.272	-0.941	0.134	1.703	1	0.192	-0.580	-0.119
trial nr.:session nr.	-0.854	0.354	-1.499	-0.166			[(2)]	-0.996	-0.621
trial nr.:group	-2.589	0.906	-4.305	-0.838			[(2)]	-3.164	-2.109
session nr.:group	-2.017	0.750	-3.510	-0.567			[(2)]	-2.416	-1.623
trial nr.:session nr.:group	-1.389	0.513	-2.375	-0.401	5.787	1	0.016	-1.670	-1.108

[(1)] trial number and session number were z-transformed to a mean of zero and a standard deviation of one, mean (sd) of the original variable were 10.5 (5.766) and 30.5 (17.319), respectively; group was dummy coded with visual=0 and visuo-spatial=1

[(2)] not indicated because of having a very limited interpretation

Table SI 6 Results of the fixed effects part of the full model of anticipatory errors in the shifted reversal experiment (model 3; estimates together with confidence limits, significance tests, and range of estimates obtained when dropping levels of random effects one at a time).

term[(1)]	Estimate	SE	lower cl	upper cl	c2	df	P	min	max
intercept	-2.266	1.105	-4.517	-0.163			[(2)]	-3.138	-1.445
trial nr.	0.662	0.352	-0.002	1.554			[(2)]	0.120	0.886
session nr.	-0.308	0.212	-0.882	0.117			[(2)]	-0.463	-0.204
session nr.^2	0.377	0.311	-0.306	0.998			[(2)]	0.057	0.577
group	-2.875	1.549	-6.573	0.084			[(2)]	-3.639	-2.061
z.Age	-0.120	0.351	-0.962	0.665	0.131	1	0.717	-0.416	0.222
SexM	-0.705	0.733	-2.552	0.812	0.980	1	0.322	-1.544	0.152
trial nr.:session nr.	-0.142	0.263	-0.722	0.424			[(2)]	-0.314	0.059
trial nr.:session nr.^2	-0.029	0.230	-0.523	0.526			[(2)]	-0.103	0.184
trial nr.:group	-0.477	0.541	-1.639	1.044			[(2)]	-0.846	0.179
session nr.:group	-0.178	0.355	-1.573	0.632			[(2)]	-0.367	-0.011
session nr.^2:group	0.261	0.477	-1.065	1.251			[(2)]	-0.049	0.596
trial nr.:session nr.:group	-0.007	0.419	-1.003	0.998	0.000	1	0.987	-0.275	0.225
trial nr.:session nr.^2:group	0.080	0.386	-0.791	1.044	0.043	1	0.837	-0.213	0.310

[(1)] trial number and session number were z-transformed to a mean of zero and a standard deviation of one, mean (sd) of the original variable were 11.75 (7.531) and 4.5 (2.292), respectively; group was dummy coded with visual=0 and visuo-spatial=1

[(2)] not indicated because of having a very limited interpretation

Table SI 7 Results of the fixed effects part of the model of anticipatory errors in the shifted reversal experiment (model 3) after removal the non-significant highest order interactions (estimates together with confidence limits and significance tests).

term[1]	Estimate	SE	lower cl	upper cl	c2	df	P
intercept	-2.278	1.101	-4.604	-0.321			[2]
trial nr.	0.641	0.335	-0.039	1.393			[2]
session nr.	-0.309	0.214	-0.941	0.090			[2]
session nr.^2	0.364	0.306	-0.295	1.006			[2]
group	-2.850	1.538	-6.738	-0.088			[2]
z.Age	-0.118	0.350	-0.931	0.758	0.131	1	0.717
SexM	-0.711	0.733	-2.439	0.969	1.017	1	0.313
trial nr.:session nr.	-0.162	0.250	-0.768	0.353			[2]
trial nr.:session nr.^2	-0.007	0.207	-0.467	0.527	0.001	1	0.971
trial nr.:group	-0.395	0.364	-1.266	0.707			[2]
session nr.:group	-0.180	0.352	-1.773	0.606			[2]
session nr.^2:group	0.291	0.453	-0.866	1.238	0.411	1	0.521
trial nr.:session nr.:group	0.023	0.395	-1.088	1.018	0.003	1	0.954

[1] trial number and session number were z-transformed to a mean of zero and a standard deviation of one, mean (sd) of the original variable were 11.75 (7.531) and 4.5 (2.292), respectively; group was dummy coded with visual=0 and visuo-spatial=1

[2] not indicated because of having a very limited interpretation

Table SI 8 Results of the fixed effects part of the model of anticipatory errors in the shifted reversal experiment (model 3) after removal of non-significant intermediate order interactions (estimates together with confidence limits and significance tests).

term[1]	Estimate	SE	lower cl	upper cl	c2	df	P
intercept	-2.556	0.952	-4.497	-0.365			[2]
trial nr.	0.676	0.222	0.221	1.250			[2]
session nr.	-0.338	0.208	-0.942	0.091			[2]
group	-2.141	0.865	-5.036	-0.322			[2]
session nr.^2	0.452	0.238	-0.080	0.921	3.710	1	0.054
z.Age	-0.117	0.341	-0.964	0.755	0.085	1	0.770
SexM	-0.710	0.711	-2.471	0.960	1.002	1	0.317
trial nr.:session nr.	-0.167	0.204	-0.663	0.302	0.681	1	0.409
trial nr.:group	-0.486	0.310	-1.220	0.475	1.970	1	0.160
session nr.:group	-0.135	0.330	-1.223	0.556	0.137	1	0.711

[1] trial number and session number were z-transformed to a mean of zero and a standard deviation of one, mean (sd) of the original variable were 11.75 (7.531) and 4.5 (2.292), respectively; group was dummy coded with visual=0 and visuo-spatial=1

[2] not indicated because of having a very limited interpretation

Table SI 9 Results of the fixed effects part of the model of anticipatory errors in the shifted reversal experiment (model 3) after removal of all non-significant interactions (estimates together with confidence limits and significance tests).

term[1]	Estimate	SE	lower cl	upper cl	c2	df	P
intercept	-2.127	0.891	-4.065	-0.194			[2]
trial nr.	0.588	0.203	0.154	1.143	7.436	1	0.006
group	-2.709	0.621	-4.600	-1.402	13.396	1	0.000
session nr.	-0.358	0.175	-0.821	0.012	4.576	1	0.032
session nr.^2	0.427	0.221	-0.105	0.917	4.092	1	0.043
z.Age	-0.107	0.335	-0.898	0.799	0.093	1	0.760
SexM	-0.683	0.711	-2.419	0.860	0.920	1	0.337

[1] trial number and session number were z-transformed to a mean of zero and a standard deviation of one, mean (sd) of the original variable were 11.75 (7.531) and 4.5 (2.292), respectively; group was dummy coded with visual=0 and visuo-spatial=1

[2] not indicated because of having a very limited interpretation

Table SI 10 Results of the fixed effects part of the full model of perseverative errors in the shifted reversal experiment (model 4; estimates together with confidence limits, significance tests, and range of estimates obtained when dropping levels of random effects one at a time).

term[1]	Estimate	SE	lower cl	upper cl	c2	df	P	min	max
intercept	-3.219	0.318	-4.014	-2.725			[2]	-3.802	-2.854
trial nr.	-2.292	0.276	-2.967	-1.819			[2]	-2.827	-2.049
session nr.	0.439	0.252	-0.047	0.992			[2]	0.330	0.567
group	-7.091	1.424	-11.001	-4.920			[2]	-12.071	-6.095
z.Age	0.031	0.181	-0.286	0.361	0.029	1	0.864	-0.075	0.131
SexM	-0.557	0.345	-1.245	0.073	2.562	1	0.109	-0.898	-0.147
trial nr.:session nr.	0.315	0.244	-0.143	0.858			[2]	0.178	0.439
trial nr.:group	-5.437	1.124	-8.375	-3.609			[2]	-9.347	-4.642
session nr.:group	-4.515	1.125	-7.206	-2.382			[2]	-6.341	-4.051
trial nr.:session nr.:group	-3.549	0.896	-5.610	-1.879	17.594	1	0.000	-4.852	-3.172

[1] trial number and session number were z-transformed to a mean of zero and a standard deviation of one, mean (sd) of the original variable were 29.25 (7.531) and 4.5 (2.292), respectively; group was dummy coded with visual=0 and visuo-spatial=1

[2] not indicated because of having a very limited interpretation

Table SI 11 Results of the random effects part of the full model of anticipatory errors in the midsession reversal experiment (model 1).

grp	effect 1[1, 2]	effect 2[2]	sd or corr
sessionID	trial nr.		0.000
sessionID	intercept		0.504
bird ID	intercept		0.919
bird ID	trial nr.		0.228
bird ID	session nr.		0.335
bird ID	session nr.^2		0.183
bird ID	trial nr.:session nr.		0.185
bird ID	trial nr.:session nr.^2		0.136
bird ID	intercept	trial nr.	-0.588
bird ID	intercept	session nr.	0.515
bird ID	intercept	session nr.^2	-0.470
bird ID	intercept	trial nr.:session nr.	-0.584
bird ID	intercept	trial nr.:session nr.^2	0.385
bird ID	trial nr.	session nr.	-0.280
bird ID	trial nr.	session nr.^2	0.102
bird ID	trial nr.	trial nr.:session nr.	0.088
bird ID	trial nr.	trial nr.:session nr.^2	-0.676
bird ID	session nr.	session nr.^2	-0.232
bird ID	session nr.	trial nr.:session nr.	-0.490
bird ID	session nr.	trial nr.:session nr.^2	0.006
bird ID	session nr.^2	trial nr.:session nr.	0.458
bird ID	session nr.^2	trial nr.:session nr.^2	-0.159
bird ID	trial nr.:session nr.	trial nr.:session nr.^2	0.471

[1] intercept indicates a random intercepts effect, all others a random slope (when column effect 2 is empty) or a correlation parameter (otherwise)

[2] trial number and session number were z-transformed to a mean of zero and a standard deviation of one, mean (sd) of the original variable were 10.5 (5.766) and 30.5 (17.319), respectively

Table SI 12 Results of the random effects part of the full model of anticipatory errors in the midsession reversal experiment (model 2).

grp	effect 1[1, 2]	effect 2[2]	sd or corr
sessionID	trial nr.		0.000
sessionID	intercept		0.509
bird ID	intercept		2.215
bird ID	trial nr.		1.537
bird ID	session nr.		1.256
bird ID	trial nr.:session nr.		0.858
bird ID	intercept	trial nr.	0.990
bird ID	intercept	session nr.	0.938
bird ID	intercept	trial nr.:session nr.	0.975
bird ID	trial nr.	session nr.	0.948
bird ID	trial nr.	trial nr.:session nr.	0.984
bird ID	session nr.	trial nr.:session nr.	0.989

[1] intercept indicates a random intercepts effect, all others a random slope (when column effect 2 is empty) or a correlation parameter (otherwise)

[2] trial number and session number were z-transformed to a mean of zero and a standard deviation of one, mean (sd) of the original variable were 10.5 (5.766) and 30.5 (17.319), respectively

Table SI 13 Results of the random effects part of the full model of anticipatory errors in the midsession reversal experiment (model 3).

grp	effect 1	effect 2	sd or corr
sessionID	trial nr.		0.000
sessionID	intercept		0.736
bird ID	intercept		2.354
bird ID	trial nr.		0.524
bird ID	session nr.		0.267
bird ID	session nr.^2		0.474
bird ID	trial nr.:session nr.		0.533
bird ID	trial nr.:session nr.^2		0.345
bird ID	intercept	trial nr.	-0.509
bird ID	intercept	session nr.	0.998
bird ID	intercept	session nr.^2	-0.808
bird ID	intercept	trial nr.:session nr.	0.509
bird ID	intercept	trial nr.:session nr.^2	-0.282
bird ID	trial nr.	session nr.	-0.453
bird ID	trial nr.	session nr.^2	0.918
bird ID	trial nr.	trial nr.:session nr.	0.482
bird ID	trial nr.	trial nr.:session nr.^2	-0.683
bird ID	session nr.	session nr.^2	-0.769
bird ID	session nr.	trial nr.:session nr.	0.563
bird ID	session nr.	trial nr.:session nr.^2	-0.342
bird ID	session nr.^2	trial nr.:session nr.	0.096
bird ID	session nr.^2	trial nr.:session nr.^2	-0.337
bird ID	trial nr.:session nr.	trial nr.:session nr.^2	-0.969

(1) trial number and session number were z-transformed to a mean of zero and a standard deviation of one, mean (sd) of the original variable were 11.75 (7.531) and 4.5 (2.292), respectively

Table SI 14 Results of the random effects part of the full model of perseverative errors in the shifted reversal experiment (model 4)^

grp	effect(1)	sd
sessionID	trial nr.	0.000
sessionID	intercept	0.160
bird ID	trial nr.:session nr.	0.000
bird ID	session nr.	0.000
bird ID	trial nr.	0.242
bird ID	intercept	0.231

(1) trial number and session number were z-transformed to a mean of zero and a standard deviation of one, mean (sd) of the original variable were 29.25 (7.531) and 4.5 (2.292), respectively

Additional Figures

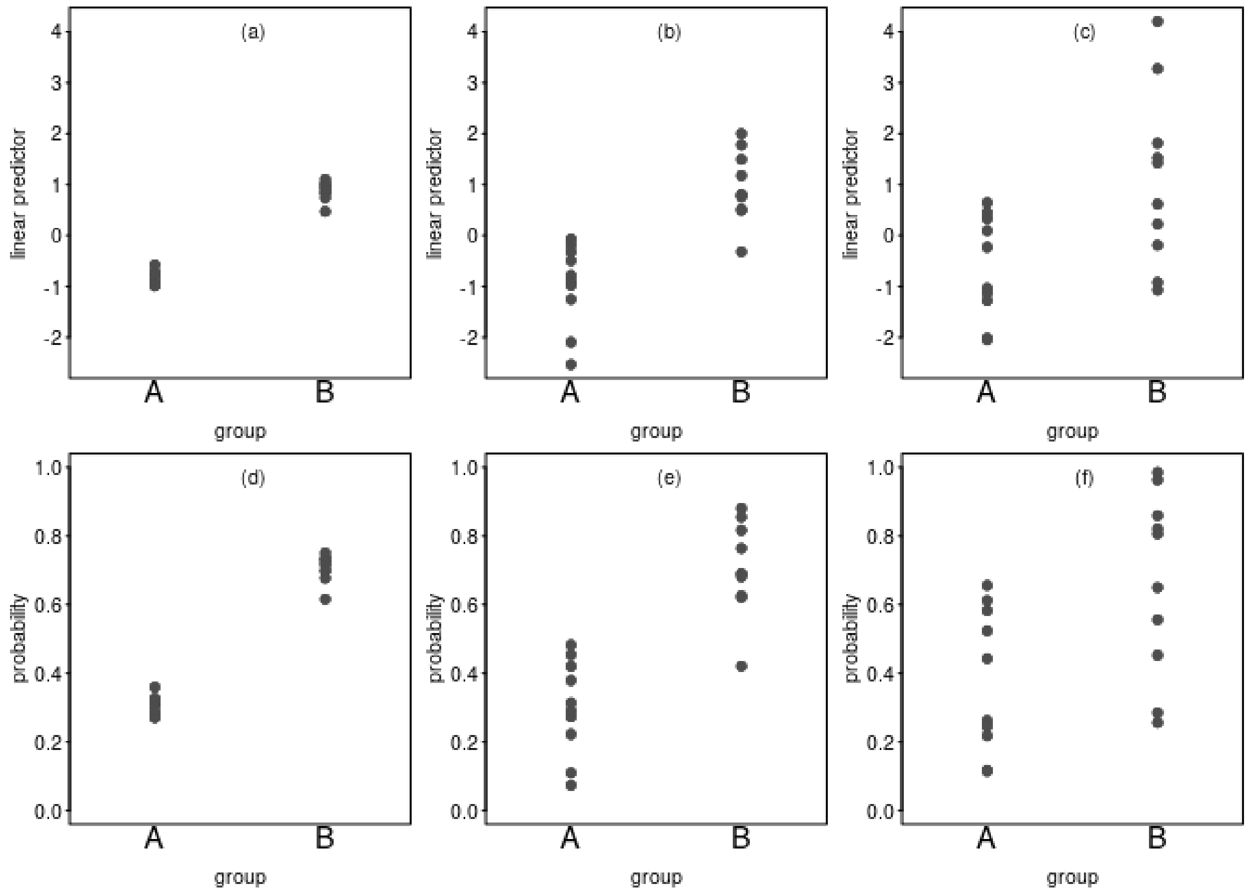

Fig. SI 1 Illustration of how one could interpret the relative contribution of a random intercepts effect using fictitious data. Shown is the effect of a fixed effects predictor which is a factor with two levels (x-axes) on a response in link space (top row) and in response (i.e., probability) space (bottom row). The fixed effect's contribution is constant throughout, but the contribution (standard deviation) of an additional random intercepts effect (which has one level for each dot) varies from 0.1 (a, d), over 0.5 (b, e), to 1 (c, f) times the fixed effects' estimate.

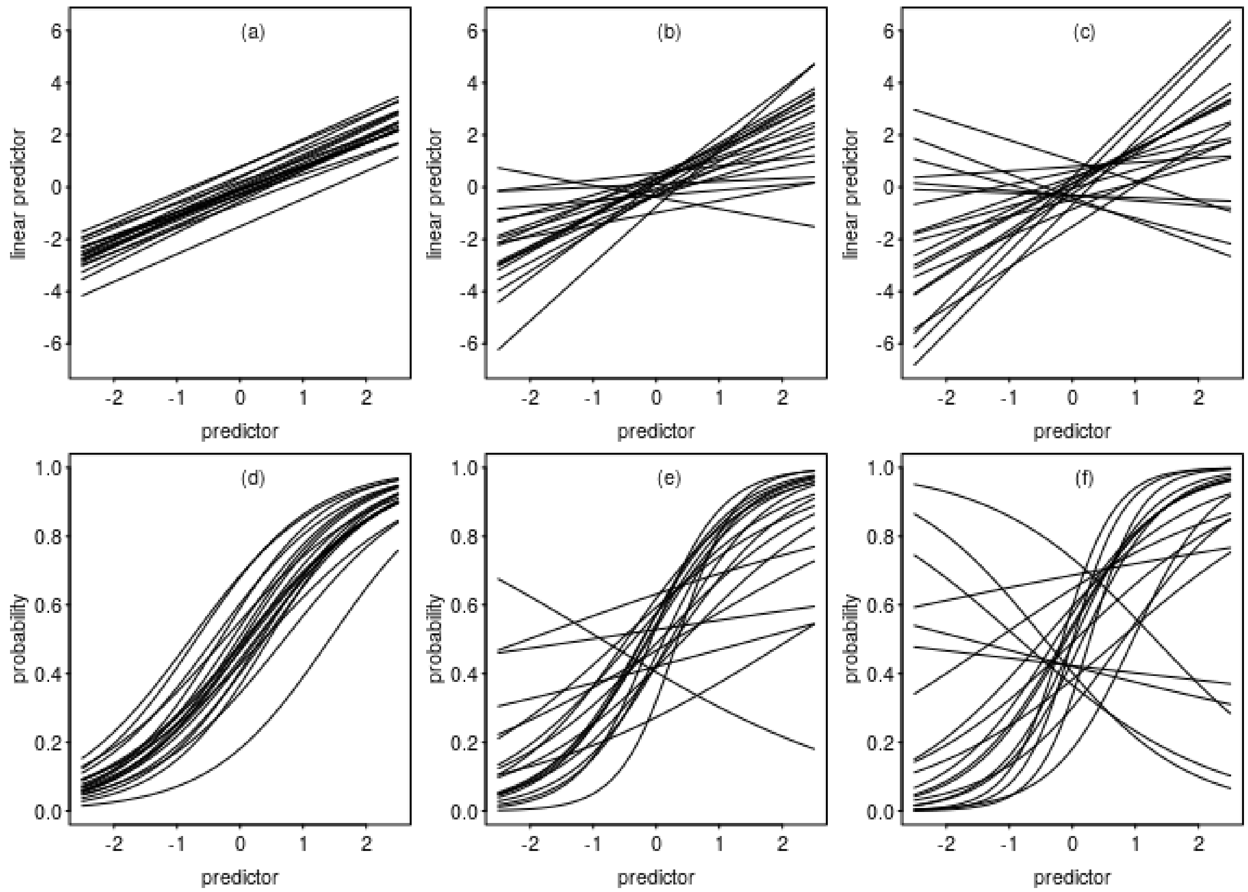

Fig. SI 2 Illustration of how one could interpret the relative contribution of a random slopes effect using fictitious data. Shown is the effect of a fixed effects predictor which is a covariate (x-axes) on a response in link space (top row) and in response (i.e., probability) space (bottom row). The fixed effect's contribution is constant throughout, but the contribution (standard deviation) of an additional random slopes effect (which is depicted with one line for each level of the random effect) varies from 0.1 (a, d), over 0.5 (b, e), to 1 (c, f) times the fixed effects' estimate.

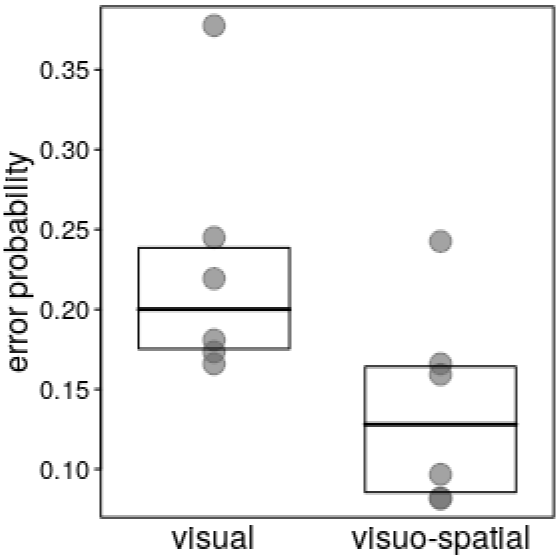

Fig. SI 3 Probability of perseverative errors (after the midsession reversal) per bird across all trials and sessions. Each dot depicts one individual (N=12) whereby darker points indicated tied observations. Vertical lines and boxes depict medians and quartiles.

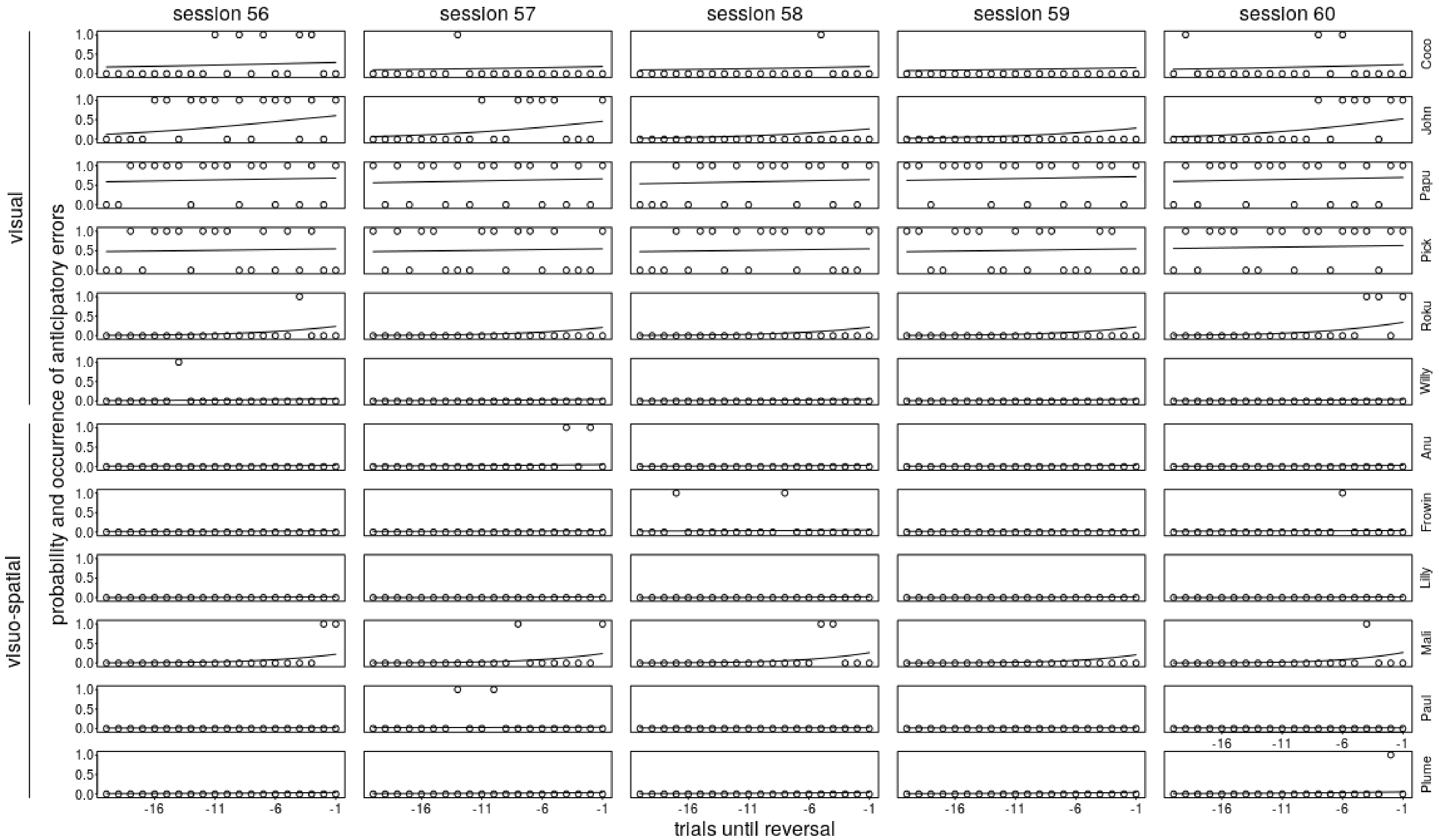

Fig. SI 4 Probability and occurrence of anticipatory errors in the last 5 sessions and for each bird in mid-session reversal experiments. Birds of the visual group are depicted in the top six rows and birds of the visuo-spatial group below. Dots show the occurrence of correct choices and errors at y-values of 0 and 1, respectively. Lines show the fitted model given the estimated fixed effects of group, trial and session number, sex, and age, and the estimated Best Linear Unbiased Predictors (BLUPs; Baayen 2008) of random intercept effects of individual and session ID as well as random slopes within these grouping variables. Note that Pick and Papu's error pattern are due to their side bias in this part of the session. Note that particularly birds of the visual groups varied considerably with regard to their overall probability of committing anticipatory errors. Note also that even within birds there was in part considerable session to session variation in the probability to commit errors (e.g., Coco and John). Furthermore, in both groups some birds seemed to tend to reversal estimation (e.g. Roku and Mali) but also not consistently so. Note also that some birds made no or only very few errors.

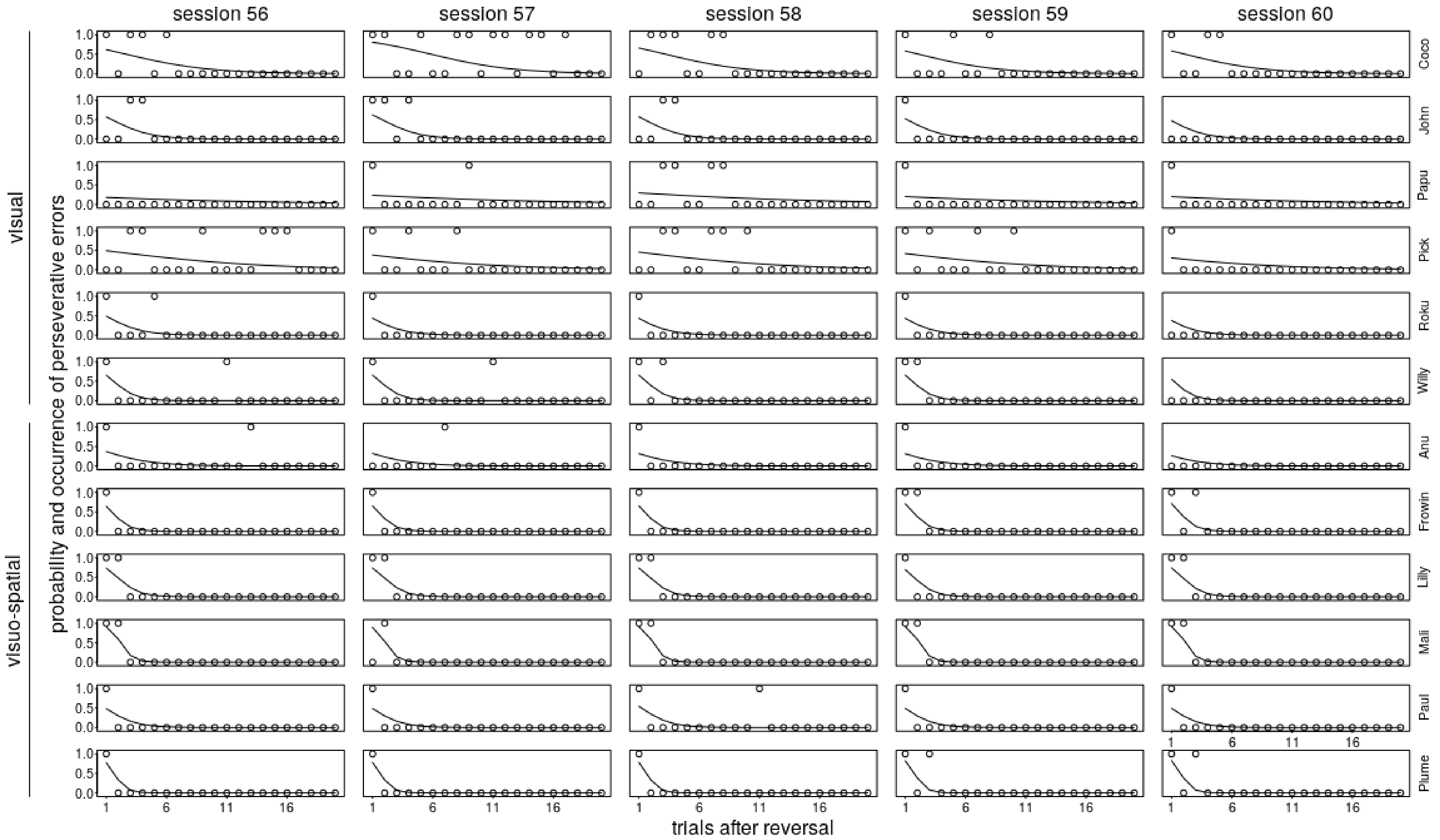

Fig. SI 5 Probability and occurrence of perseverative errors in the last 5 sessions and for each bird in mid-session reversal experiments. Birds of the visual group are depicted in the top six rows and birds of the visuo-spatial group below. Dots show the occurrence of correct choices and errors at y-values of 0 and 1, respectively. Lines show the fitted model given the estimated fixed effects of group, trial and session number, sex, and age, and the estimated BLUPs of random intercept effects of individual and session ID as well as random slopes within these grouping variables. Note that birds of the visuo-spatial groups and also some of the visual group always or mostly committed perseverative errors in only the first three trials after the reversal (and sometimes only in the very first trail after the reversal. Note also that even within birds there was in part considerable session to session variation in the probability to commit errors (e.g. Pick).

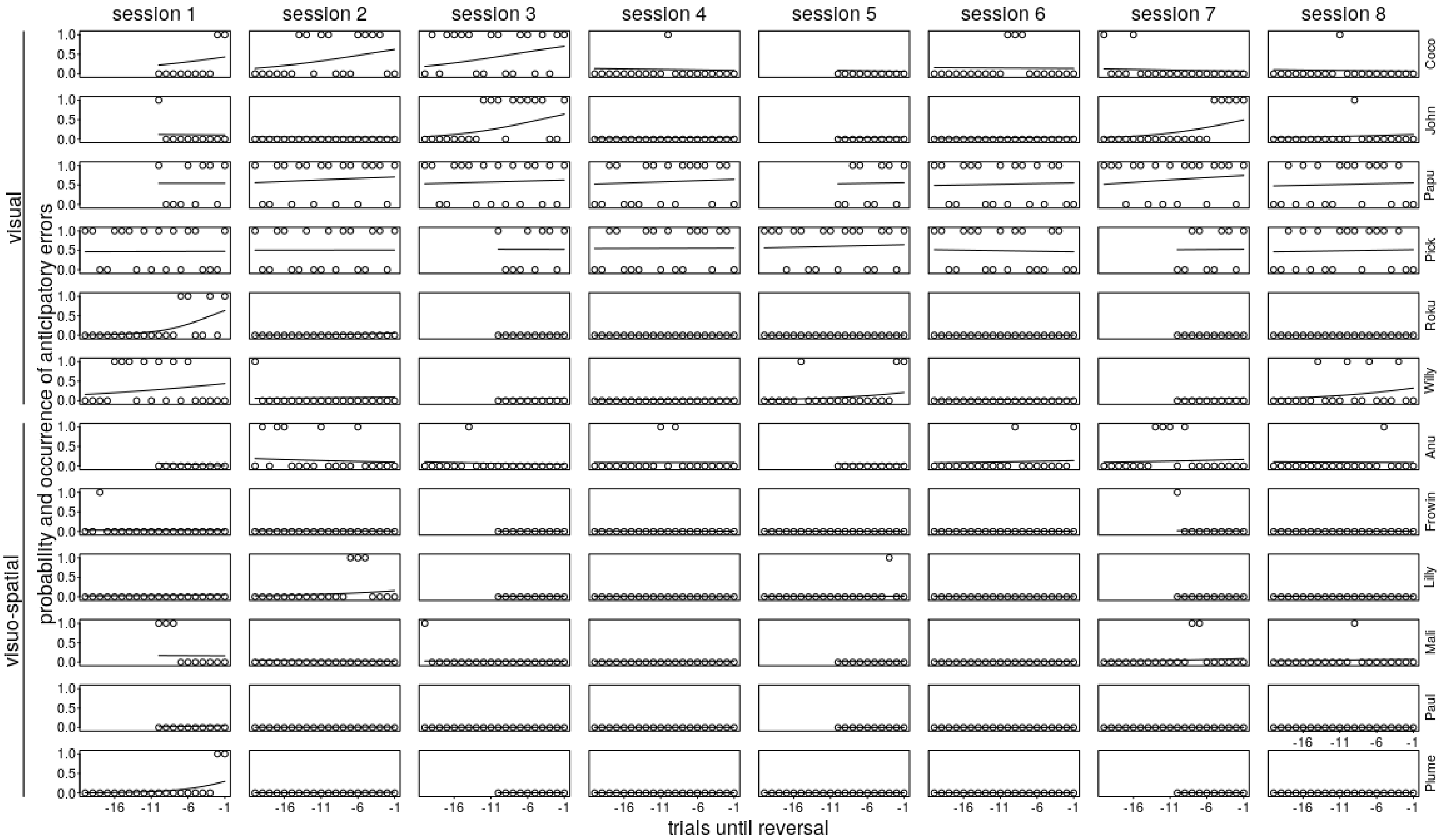

Fig. SI 6 Probability and occurrence of anticipatory errors for each bird in the 8 sessions of shifted reversal experiments. Birds of the visual group are depicted in the top six rows and birds of the visuo-spatial group below. Dots show the occurrence of correct choices and errors at y-values of 0 and 1, respectively. Lines show the fitted model given the estimated fixed effects of group, trial and session number, sex, and age, and the estimated BLUPs of random intercept effects of individual and session ID as well as random slopes within these grouping variables. Note that Pick and Papu's error pattern are due to their side bias in this part of the session. Note that the overall error probability varied considerably among birds and was relatively high in some birds of the visuo-spatial group and that some birds did not commit a single or very few anticipatory errors. Note also that even within birds there was in part considerable session to session variation in the probability to commit errors (e.g. Coco, Willy or Anu).

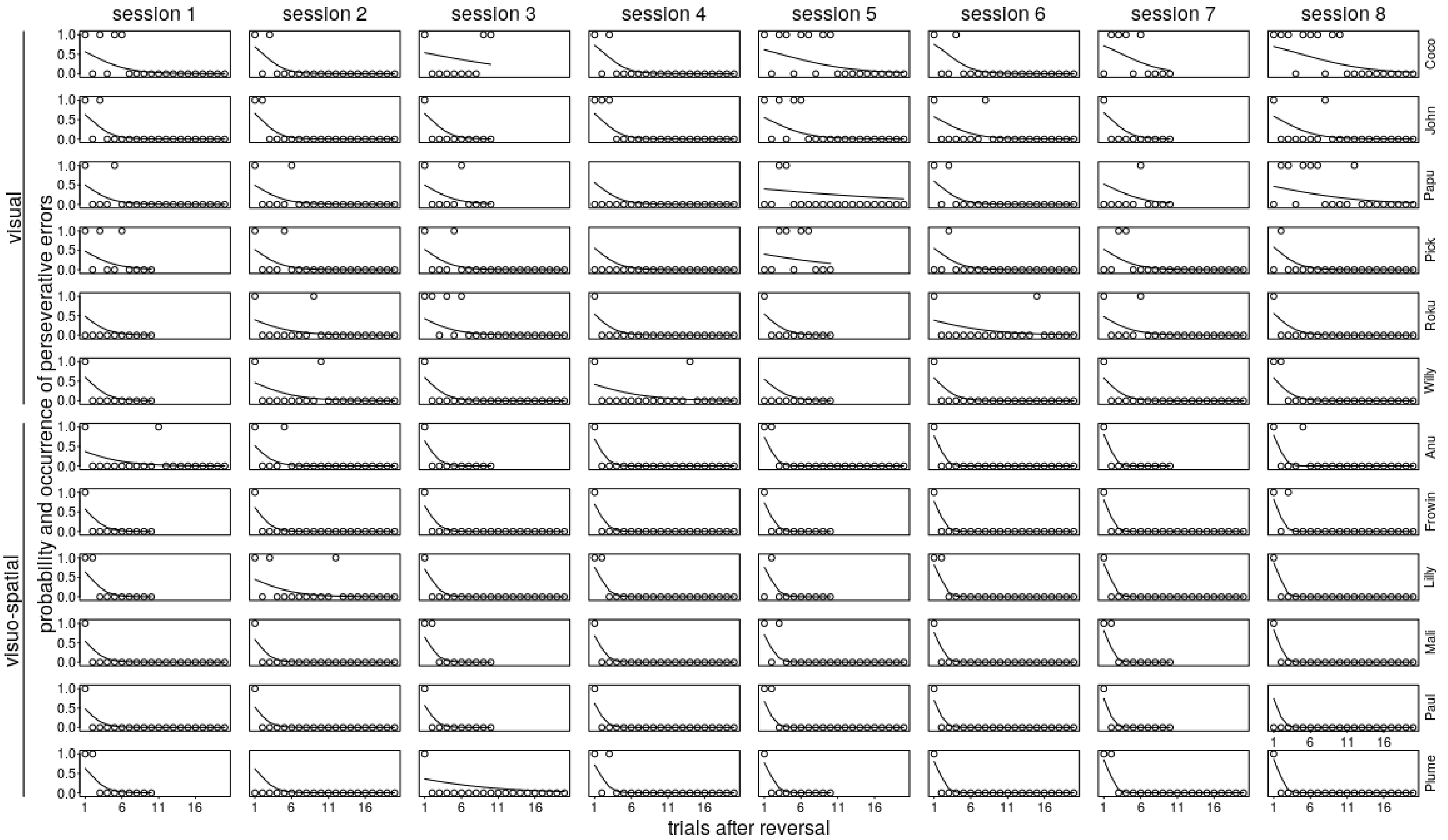

Fig. SI 7 Probability and occurrence of perseverative errors in the last 5 sessions and for each bird in shifted reversal experiments. Birds of the visual group are depicted in the top six rows and birds of the visuo-spatial group below. Dots show the occurrence of correct choices and errors at y-values of 0 and 1, respectively. Lines show the fitted model given the estimated fixed effects of group, trial and session number, sex, and age, and the estimated BLUPs of random intercept effects of individual and session ID as well as random slopes within these grouping variables. Note that birds of the visuo-spatial groups and also some of the visual group always or mostly committed perseverative errors in only the first three trials after the reversal (and sometimes only in the very first trail after the reversal.

Individual performance visual group

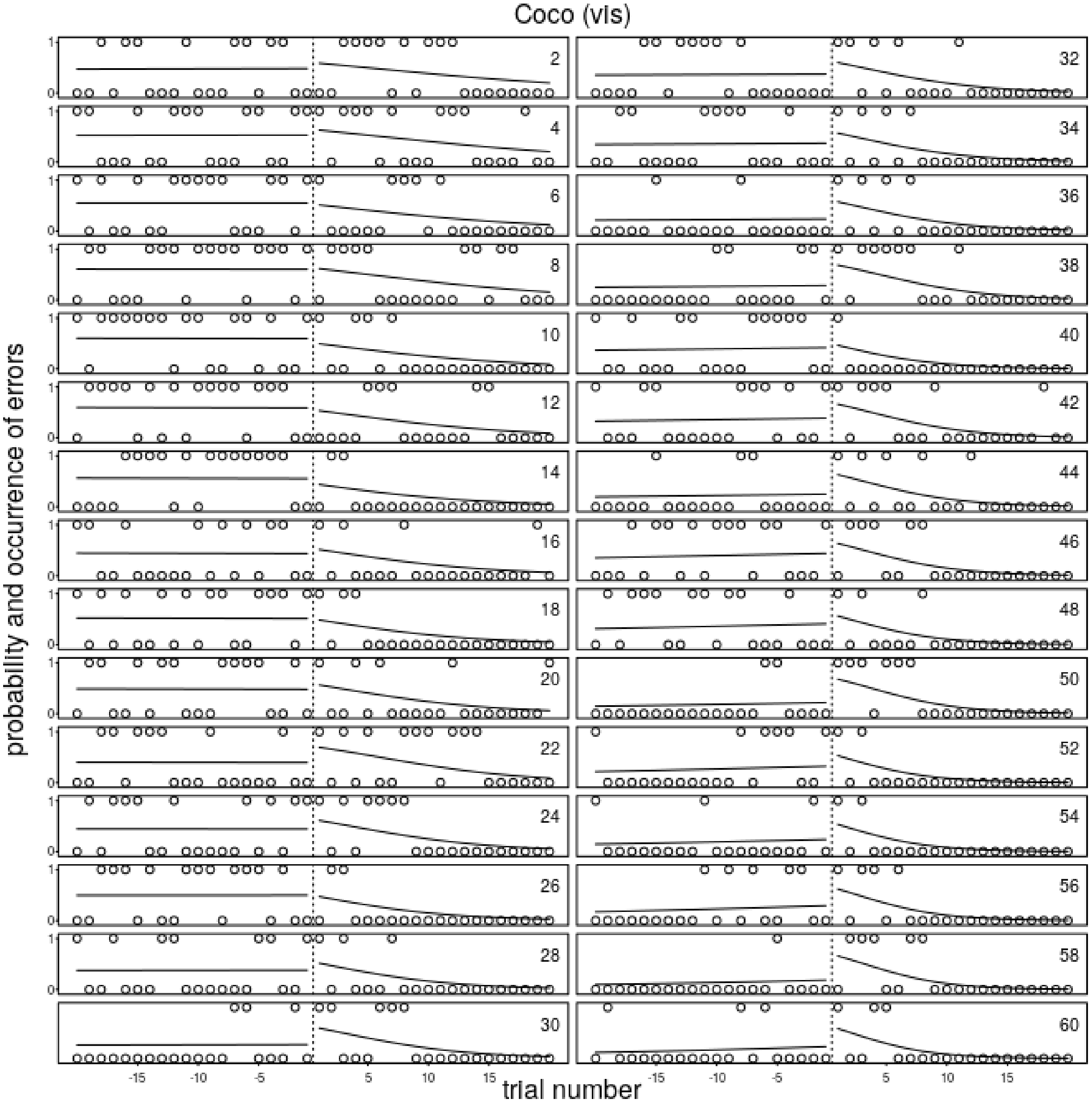

Fig. SI 8 Individual performance of the female Coco (visual group) in every even session of the midsession reversal task. Dots show the occurrence of correct choices and errors at y-values of 0 and 1, respectively. Lines show the fitted model. Coco learned her second correct stimulus first and followed both correct stimuli consistently by session 35. She started to participate in the experiment at a later point than the other birds and as a result had a tighter testing schedule which possibly influenced her motivation to fully cooperate.

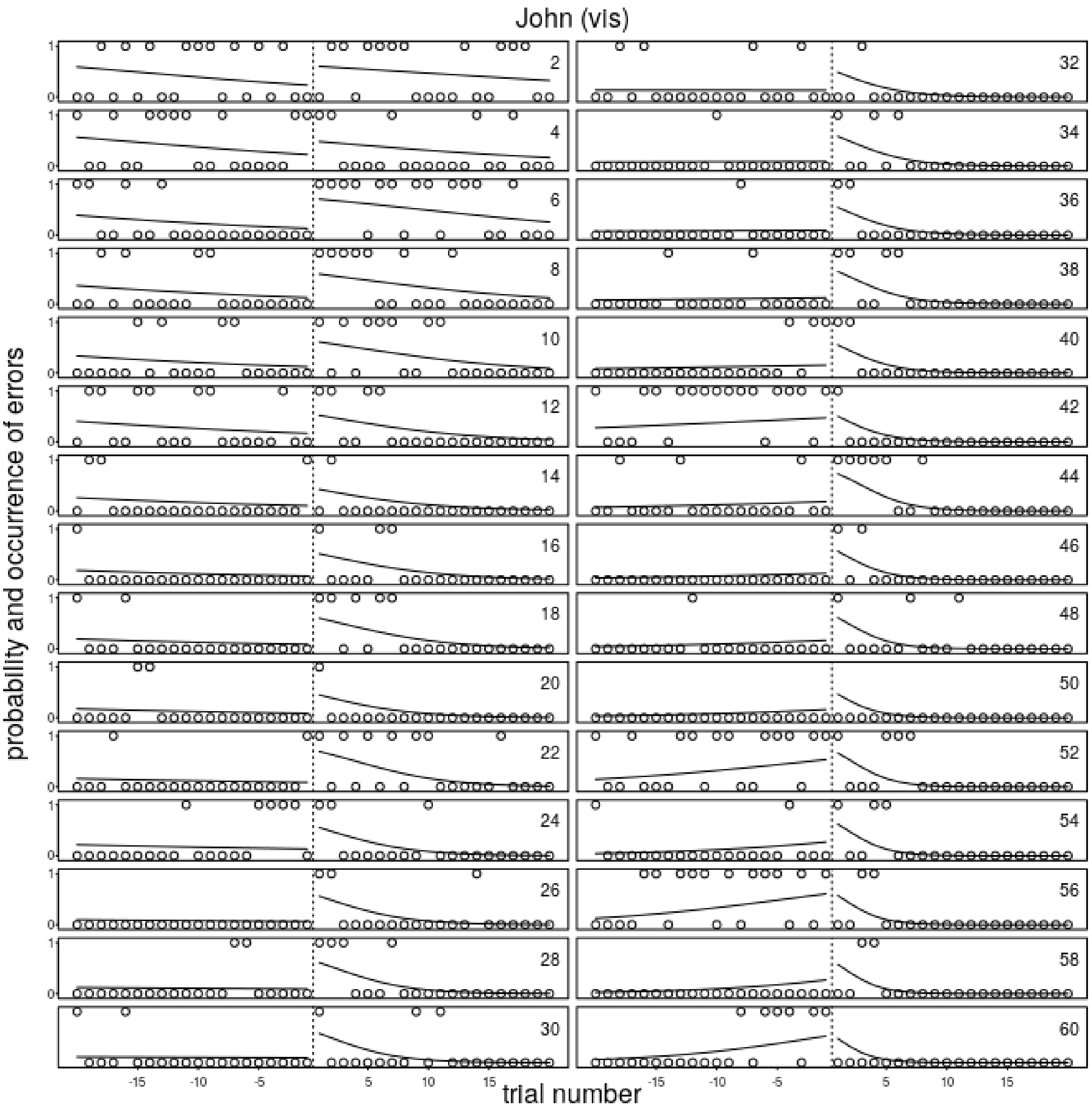

Fig. SI 9 Individual performance of the male John (visual group) in every even session of the midsession reversal task. Dots show the occurrence of correct choices and errors at y-values of 0 and 1, respectively. Lines show the fitted model. John learned his second correct stimulus first and followed both correct stimuli consistently by session 8. Although being able to show perfect win-stay/lose-shift he preferred to estimate the reversal. He is the only bird with three completely correct sessions (#41, #50, #53).

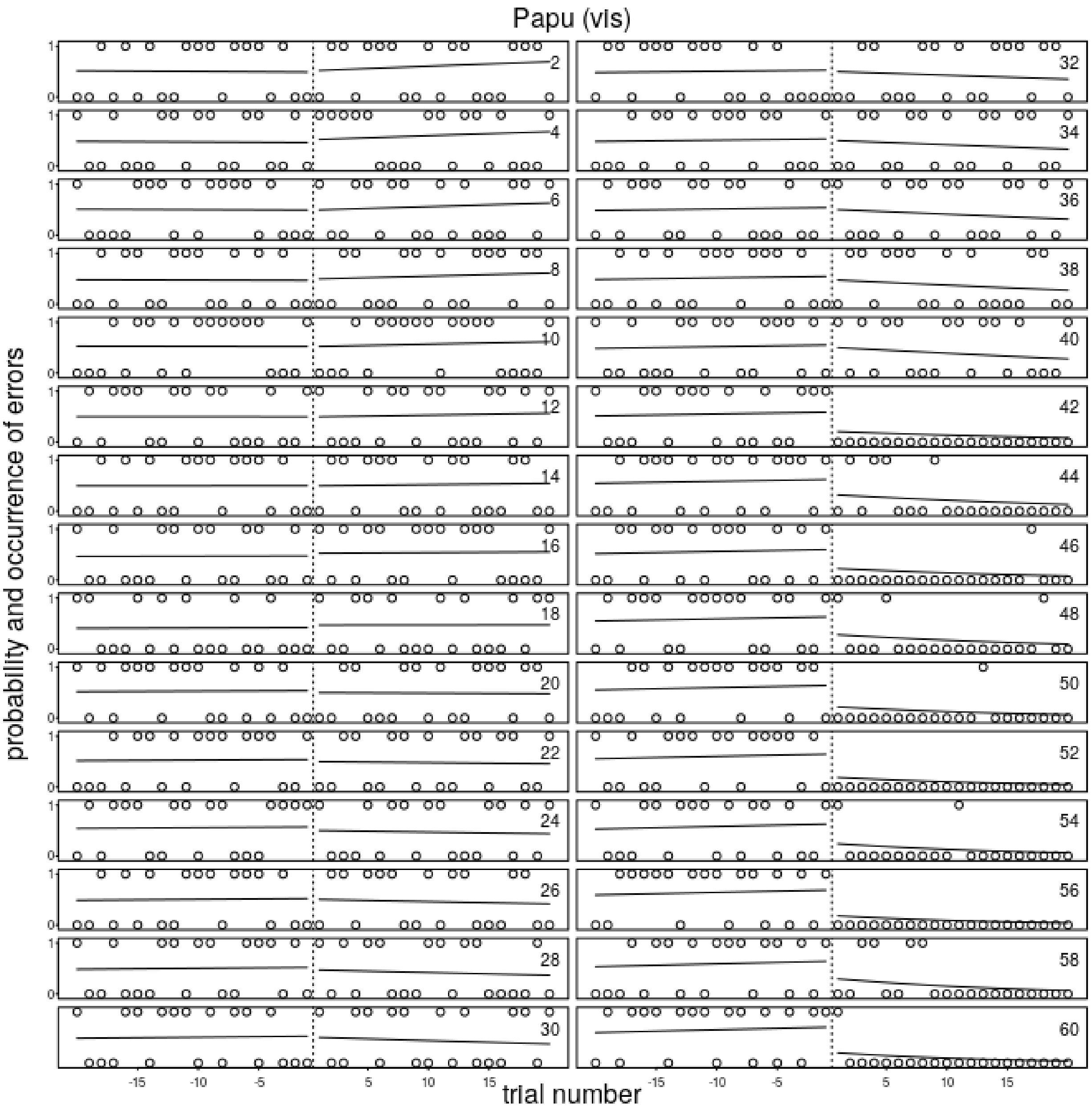

Fig. SI 10 Individual performance of the female Papu (visual group) in every even session of the midsession reversal task. Dots show the occurrence of correct choices and errors at y-values of 0 and 1, respectively. Lines show the fitted model. Papu learned her second correct stimulus by session 42, but never learned her first correct stimulus. She developed an exit strategy, a side bias, in the first half, but reacted to the reversal information and had comparable performance to her group members in the second half. There was no evidence for lack of motivation to take part in the test, but it seems possible that she was still affected by her first breeding season in the initial 30-40 sessions of testing.

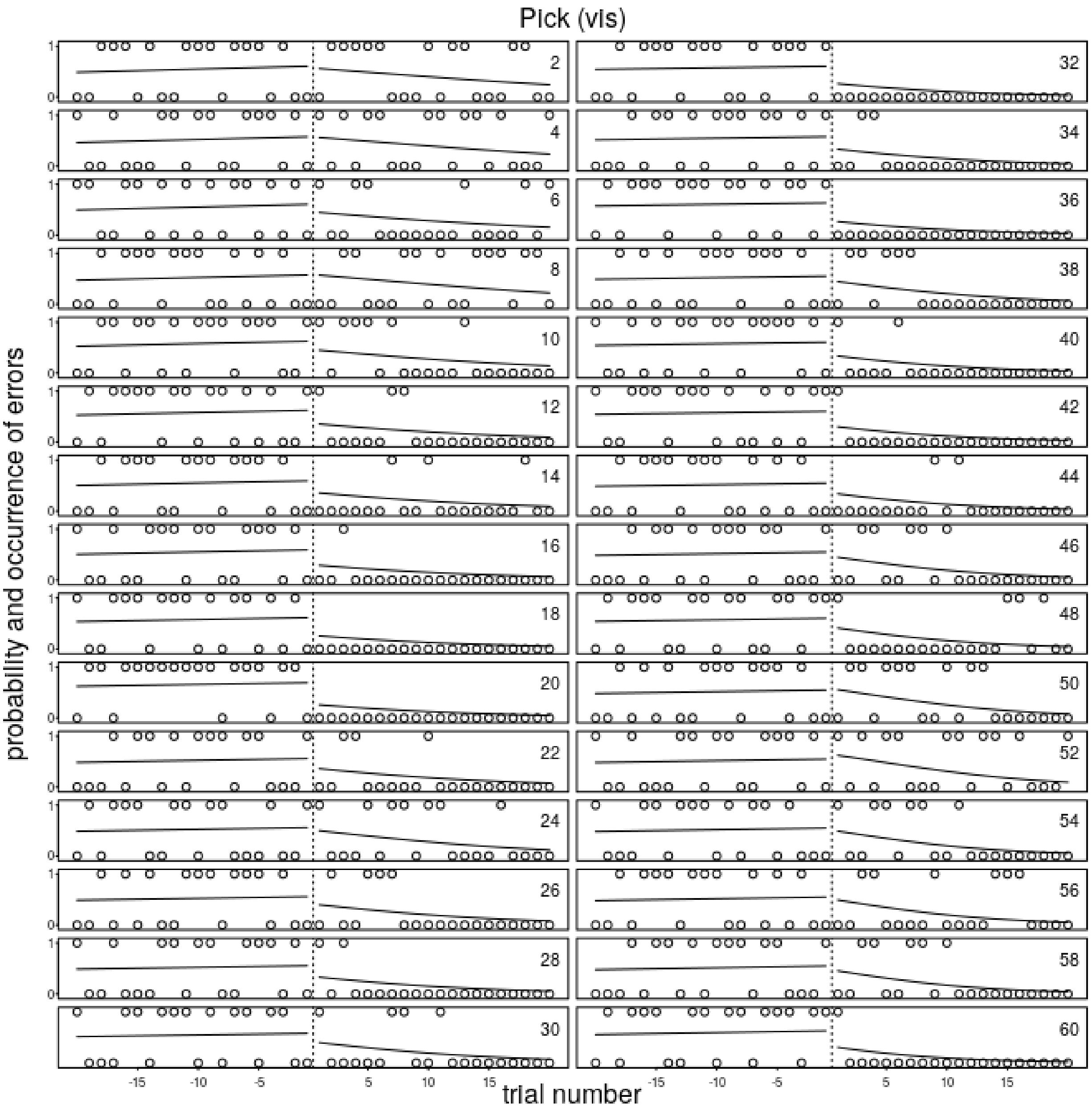

Fig. SI 11 Individual performance of the male Pick (visual group) in every even session of the midsession reversal task. Dots show the occurrence of correct choices and errors at y-values of 0 and 1, respectively. Lines show the fitted model. Pick learned his second correct stimulus by session 9, but like Papu never learned his first correct stimulus and showed the same exit strategy. Pick's and Papu's responses could possibly be explained if the birds only focused on the rule for their second stimulus and never considered the other stimulus as well.

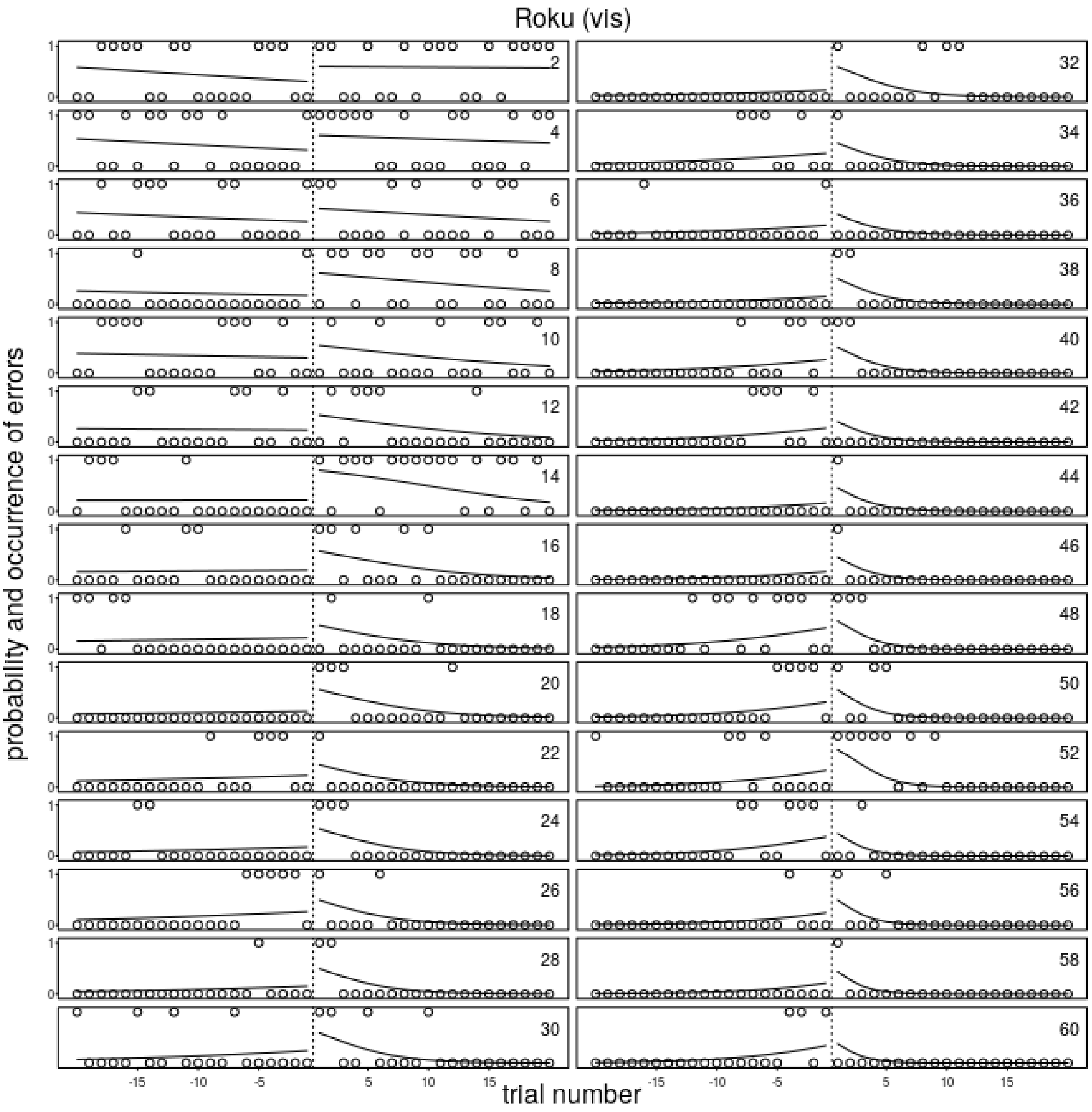

Fig. SI 12 Individual performance of the male Roku (visual group) in every even session of the midsession reversal task. Dots show the occurrence of correct choices and errors at y-values of 0 and 1, respectively. Lines show the fitted model. Roku learned his first correct stimulus first and followed both correct stimuli consistently by session 16. Although being able to show perfect win-stay/lose-shift he preferred to estimate the reversal in the midsession reversal task but shifted his preference in the shifted reversal task (not depicted) to win-stay/lose-shift.

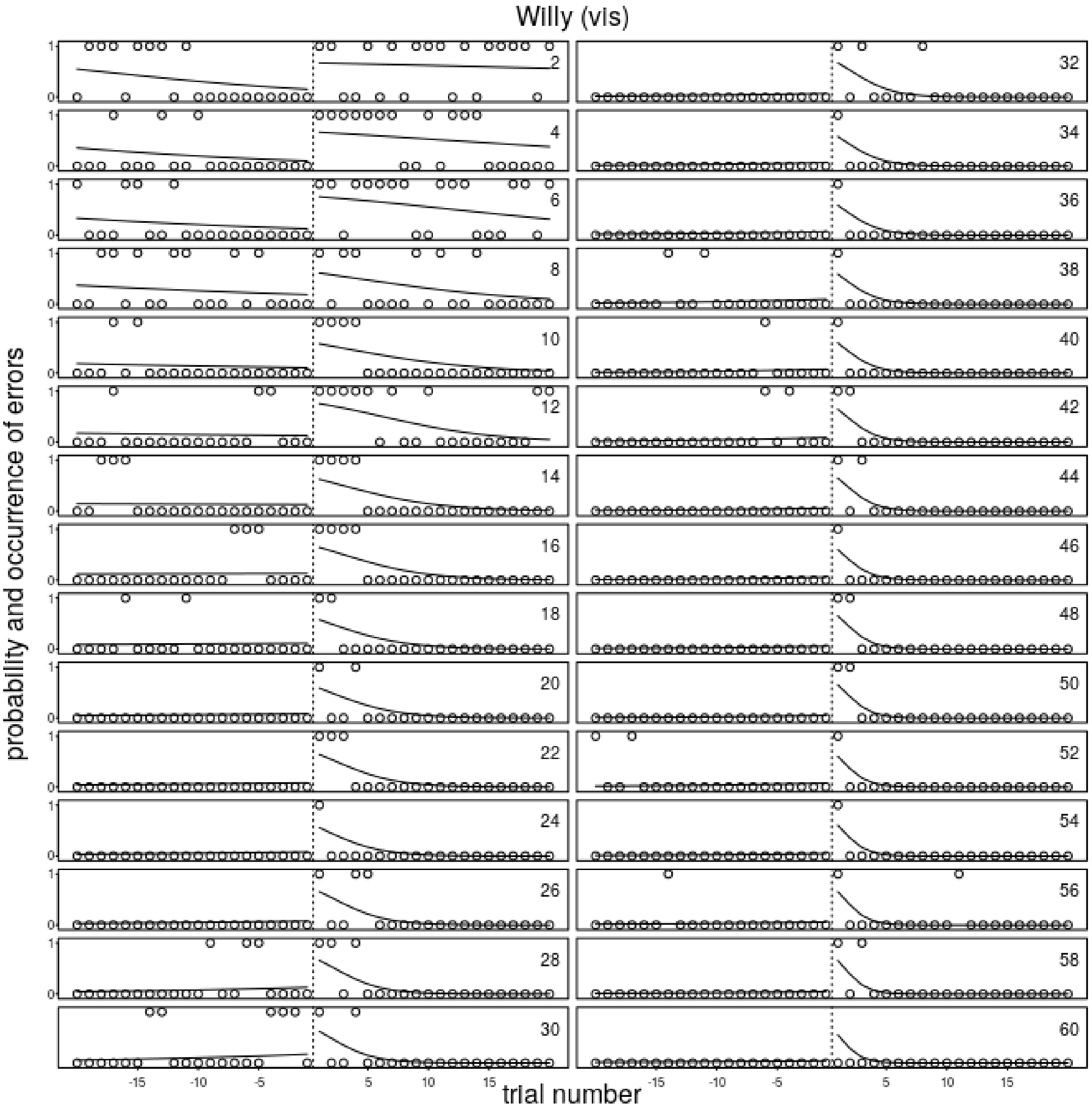

Fig. SI 13 Individual performance of the female Willy (visual group) in every even session of the midsession reversal task. Dots show the occurrence of correct choices and errors at y-values of 0 and 1, respectively. Lines show the fitted model. Willy learned her first correct stimulus first and followed both correct stimuli consistently by session 9. She was able to show perfect win-stay/lose-shift and preferred this strategy even when not always executed exact. Nevertheless, she had a completely correct session (#60). When solving tasks, Willy is said to take her time, which might have been beneficial in this setting as it enabled her to calmly orient towards the correct stimulus and therefore avoid partial reinforcement through careless mistakes.

Individual performance visuo-spatial group

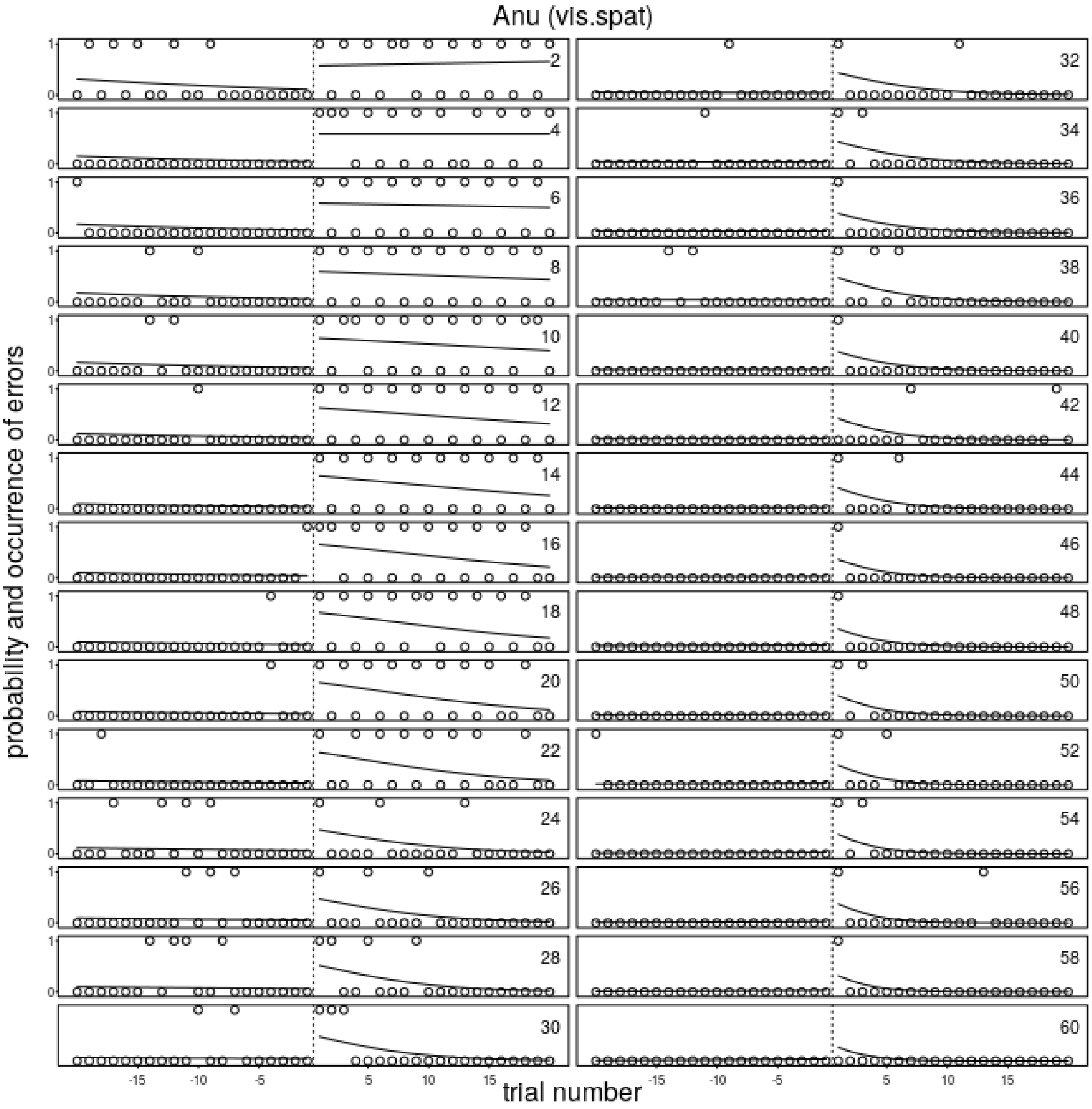

Fig. SI 14 Individual performance of the male Anu (visuo-spatial group) in every even session of the midsession reversal task. Dots show the occurrence of correct choices and errors at y-values of 0 and 1, respectively. Lines show the fitted model. Anu learned his first correct stimulus first and followed both correct stimuli consistently by session 24. He preferred win-stay/lose-shift but had a completely correct session (#60). Please also note the correctly estimated reversal in session 42.

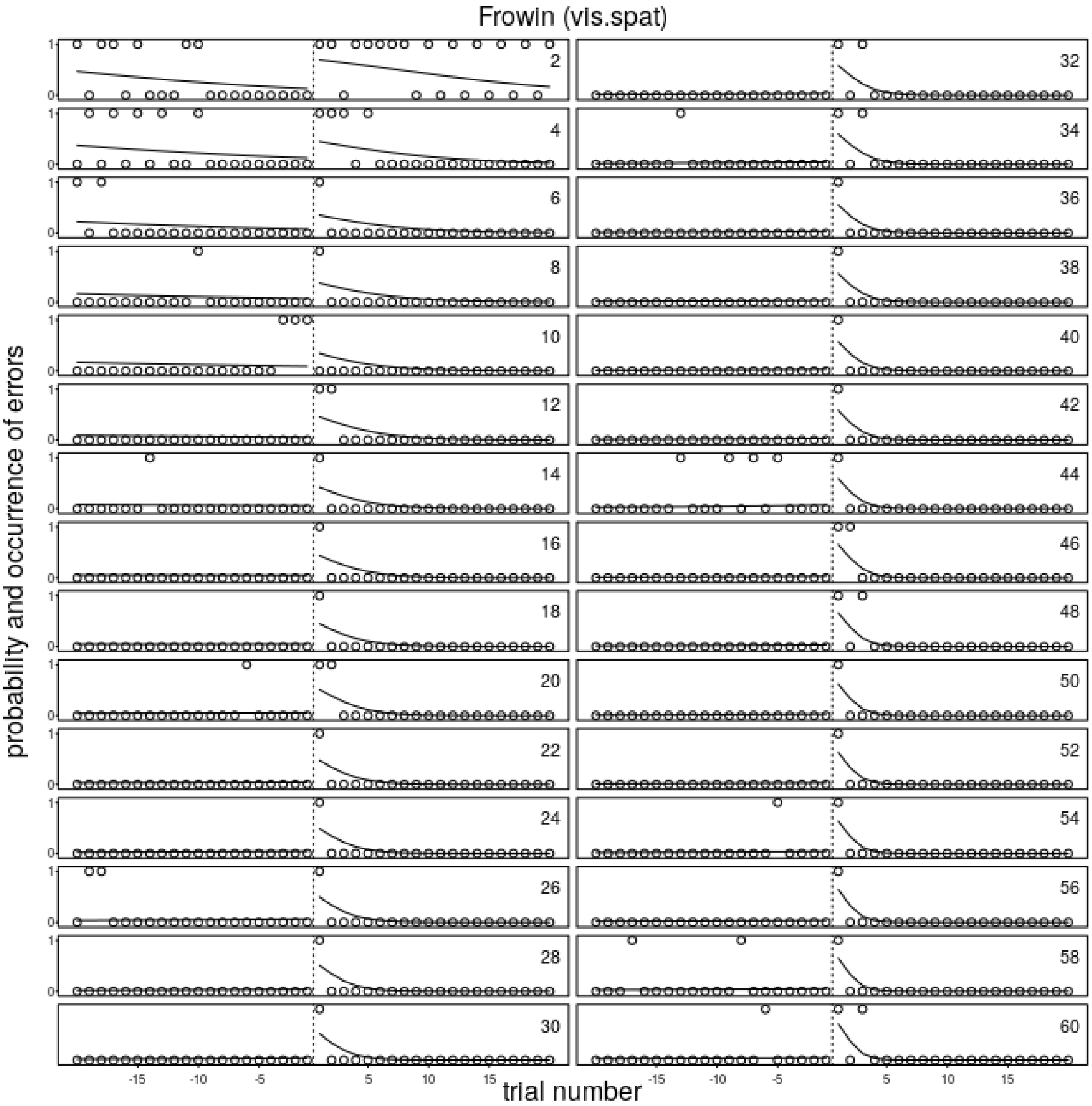

Fig. SI 15 Individual performance of the male Frowin (visuo-spatial group) in every even session of the midsession reversal task. Dots show the occurrence of correct choices and errors at y-values of 0 and 1, respectively. Lines show the fitted model. Frowin learned his second correct stimulus first and followed both correct stimuli consistently by session 3. He preferred win-stay/lose-shift but was the first bird to have a completely correct session (#19). Frowin sometimes shows a "check back" pattern, where he had already correctly shifted his responses but then is incorrect for one more trial (eg #48 or #60). Such sessions would not meet the description of win-stay/lose-shift (Cook and Rosen 2010). Errors might occur due to the birds' curiosity.

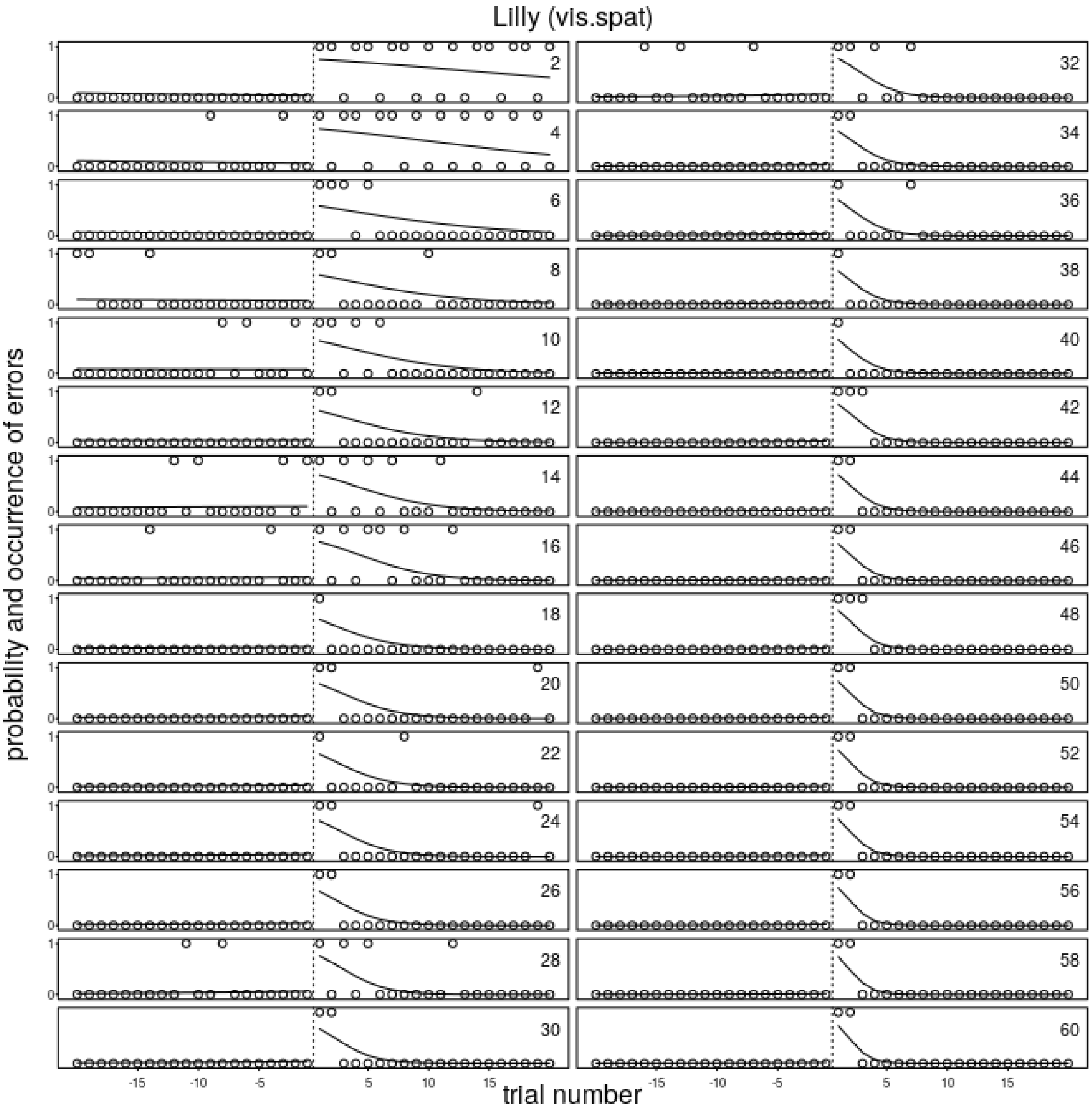

Fig. SI 16 Individual performance of the female Lilly (visuo-spatial group) in every even session of the midsession reversal task. Dots show the occurrence of correct choices and errors at y-values of 0 and 1, respectively. Lines show the fitted model. Lilly learned her first correct stimulus first and followed both correct stimuli consistently by session 6. She preferred win-stay/lose-shift but usually needed two consecutive mistakes to shift her behaviour. These sessions would meet the description of win-stay/lose-shift (Cook and Rosen 2010).

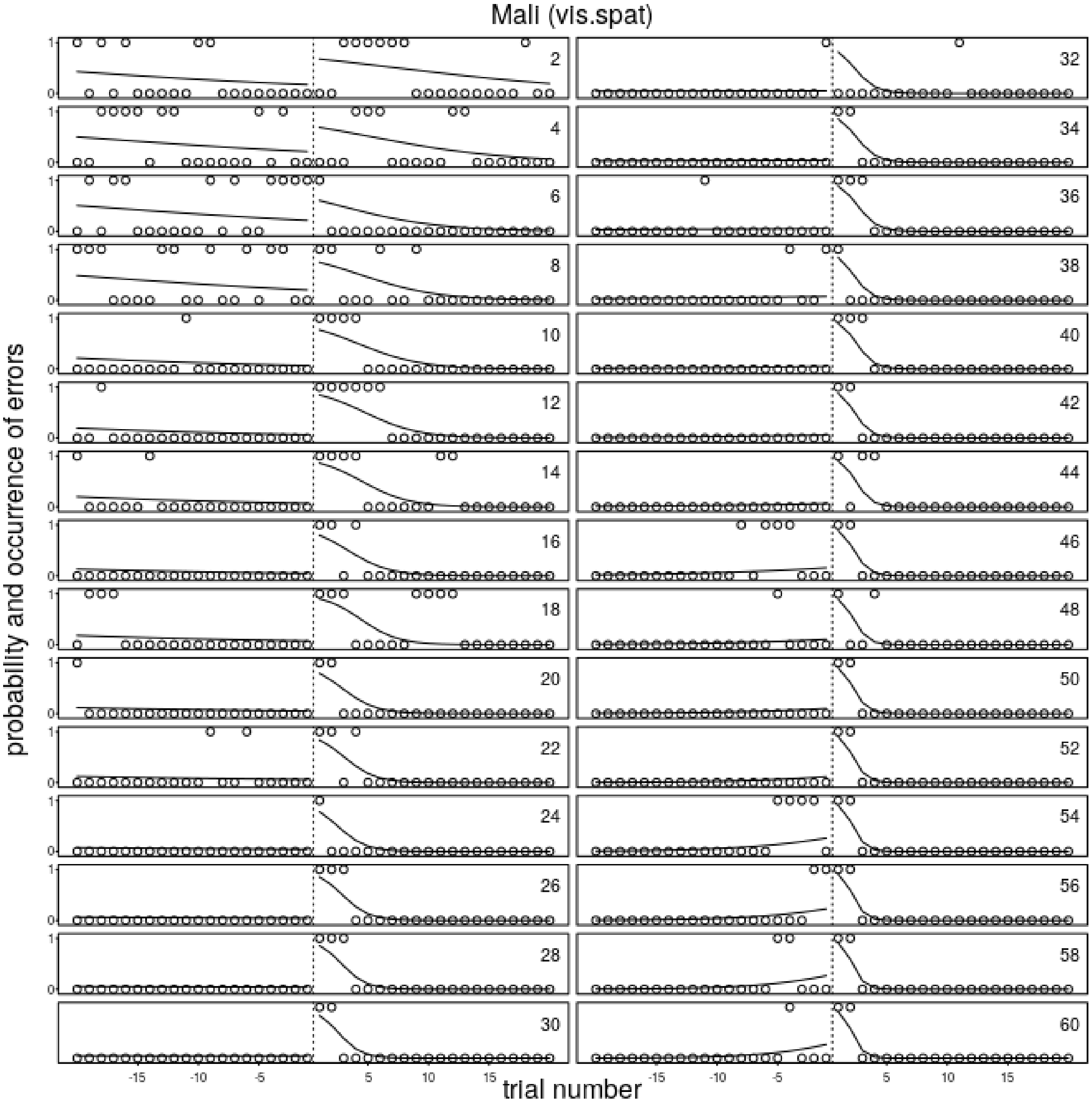

Fig. SI 17 Individual performance of the female Mali (visuo-spatial group) in every even session of the midsession reversal task. Dots show the occurrence of correct choices and errors at y-values of 0 and 1, respectively. Lines show the fitted model. Mali learned her first correct stimulus first and followed both correct stimuli consistently by session 12. This was her first participation in any kind of study and she had been trained for the touchscreen just before testing. She preferred win-stay/lose-shift but as Lilly she usually made two consecutive mistakes before shifting her behaviour.

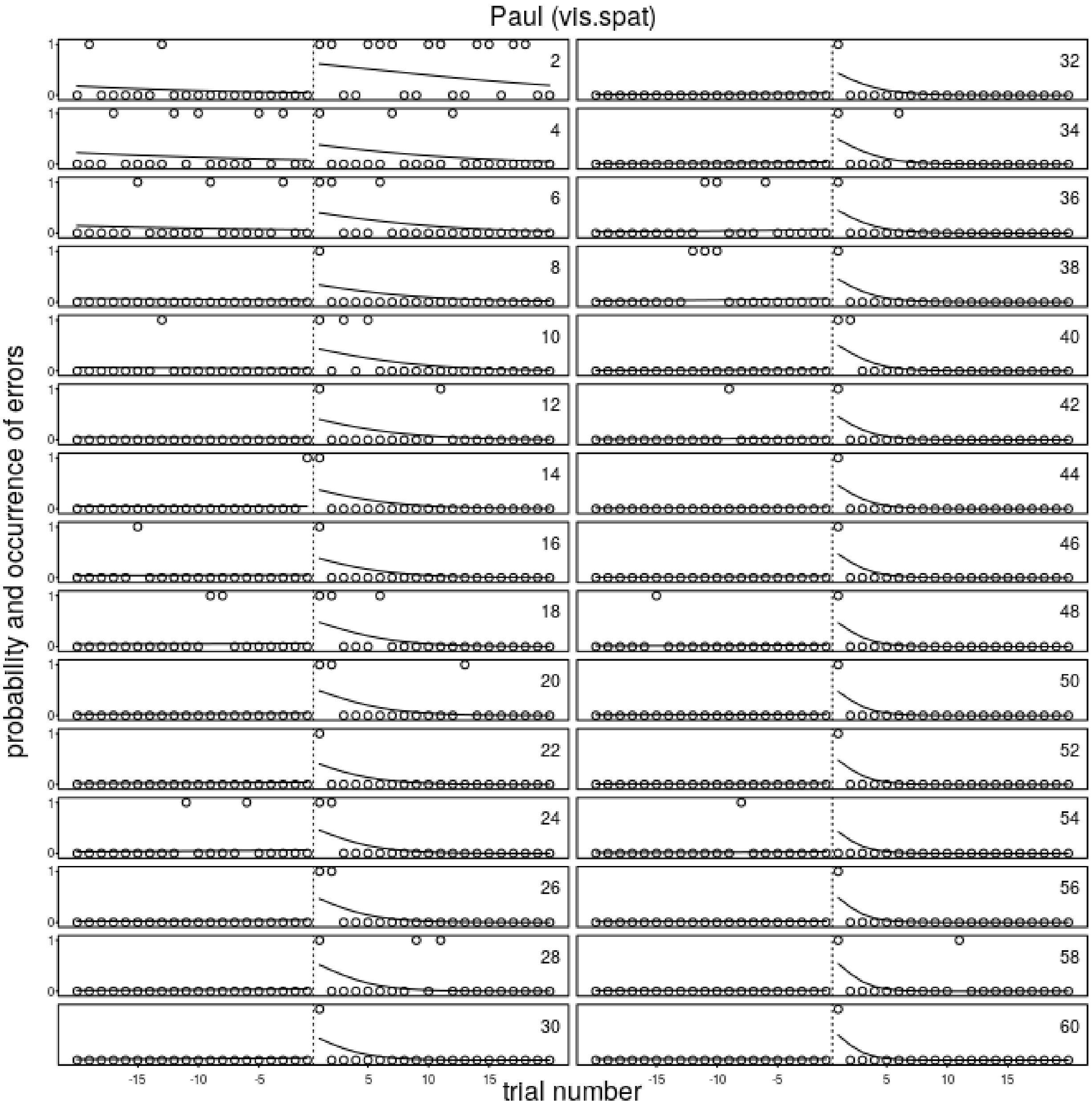

Fig. SI 18 Individual performance of the male Paul (visuo-spatial group) in every even session of the midsession reversal task. Dots show the occurrence of correct choices and errors at y-values of 0 and 1, respectively. Lines show the fitted model. Paul learned both correct stimuli in his very first session and followed them consistently by session 7. He preferred win-stay/lose-shift but was able to estimate the reversal correctly (see #54). He had one completely correct session in a middle reversal session of the shifted reversal task (#8A, not depicted).

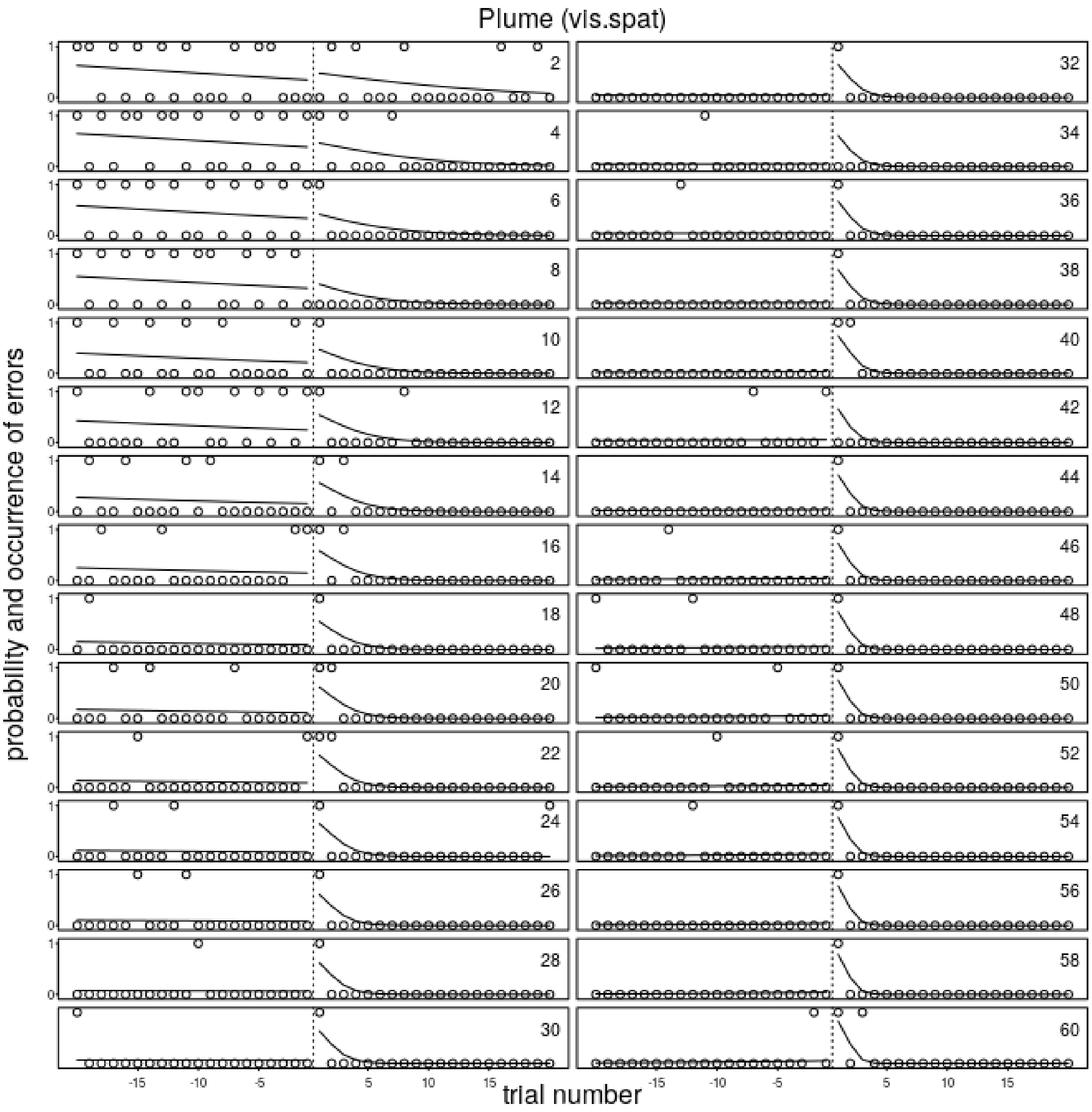

Fig. SI 19 Individual performance of the female Plume (visuo-spatial group) in every even session of the midsession reversal task. Dots show the occurrence of correct choices and errors at y-values of 0 and 1, respectively. Lines show the fitted model. Plume learned her second correct stimulus first and followed both correct stimuli consistently by session 14. She preferred win-stay/lose-shift but was able to estimate the reversal correctly (see #34). She had one completely correct session in a middle reversal session of the shifted reversal task (#2A, not depicted).

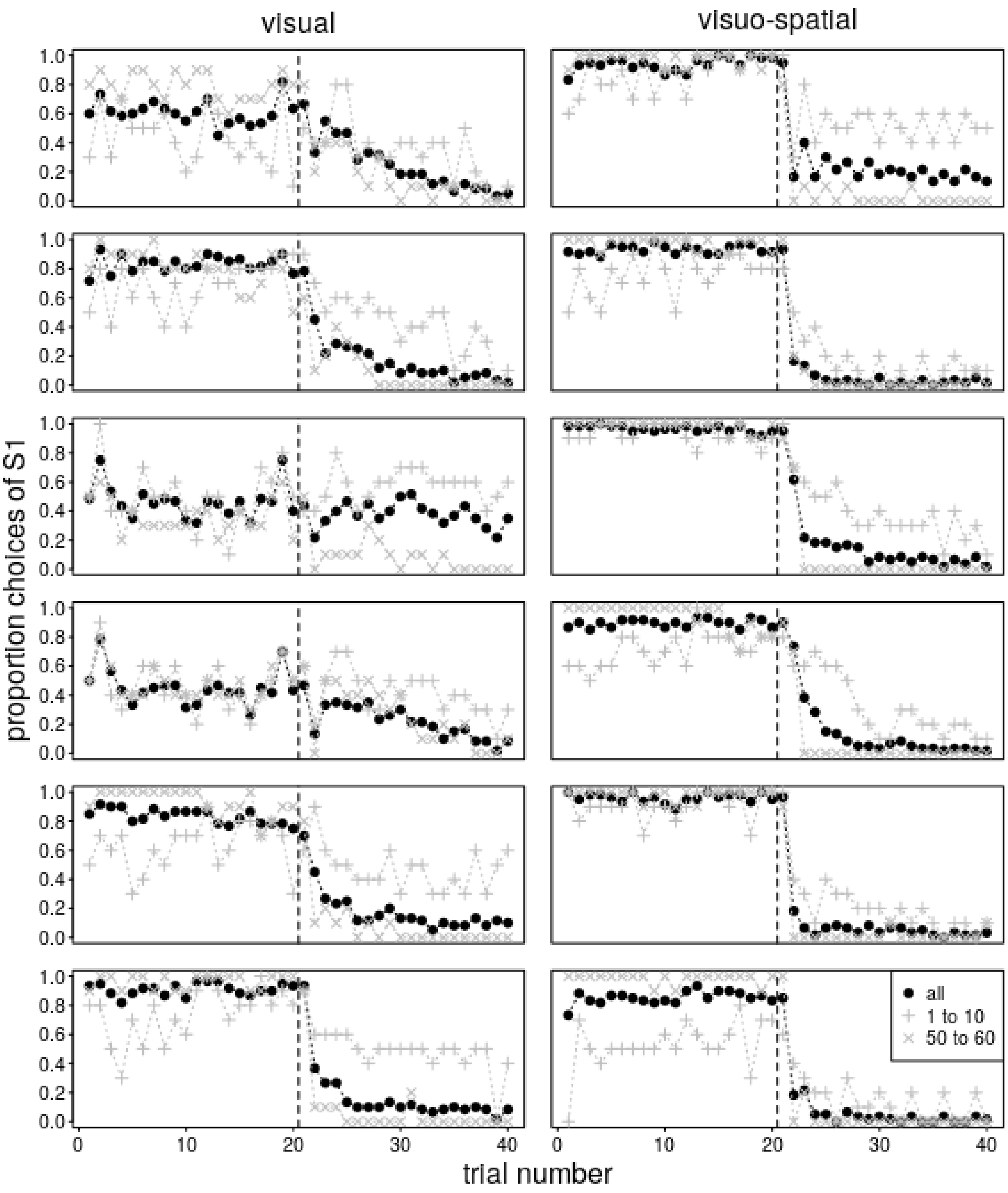

Fig. SI 20 Proportion trials in which the birds chose the first correct stimulus (S1) in the mid-session reversal experiment separately for each bird. Left column from top to bottom: Coco, John, Pick, Papu, Roku, Willy. Right column from top to bottom: Anu, Frowin, Lilly, Mali, Paul, Plume.

References

Baayen RH (2008) Analyzing linguistic data. Cambridge University Press, Cambridge

Cook RG, Rosen HA (2010) Temporal control of internal states in pigeons. Psychon Bull Rev 17:915–922. https://doi.org/10.3758/PBR.17.6.915
